# Generation and identification of a conditional knockout allele for the PSMD11 gene in mice

**DOI:** 10.1186/s12861-020-00233-1

**Published:** 2021-02-01

**Authors:** Linlin Zhao, Jinming Zhao, Yingying Zhang, Lele Wang, Longyan Zuo, Airu Niu, Wei Zhang, Xia Xue, Suhong Zhao, Chao Sun, Kailin Li, Jue Wang, Zhimin Bian, Xiaogang Zhao, Dieter Saur, Barbara Seidler, Chuanxin Wang, Tonggang Qi

**Affiliations:** 1 Institute of Medical Sciences, The Second Hospital, Cheeloo College of Medicine, Shandong University, Jinan, 250033 China; 2 Department of Clinical Laboratory, The Second Hospital, Cheeloo College of Medicine, Shandong University, Jinan, 250033 China; 3 Department of Pathology, Liaocheng People’s Hospital, Liaocheng, 252000 China; 4 Department of Clinical Laboratory, Sanhe Yanjiao No.23 Hospital, Beijing, 065201 China; 5 Department of Medical Imaging, The Second Hospital, Cheeloo College of Medicine, Shandong University, Jinan, 250033 China; 6 Department of Pharmacy, The Second Hospital, Cheeloo College of Medicine, Shandong University, Jinan, 250033 China; 7 Comprehensive Department, National Cancer Center/National Clinical Research Center for Cancer/Cancer Hospital, Chinese Academy of Medical Sciences and Peking Union Medical College, Beijing, 100021 China; 8 Department of Thoracic Surgery/Key Laboratory of Thoracic Cancer in Universities of Shandong, The Second Hospital, Cheeloo College of Medicine, Shandong University, Jinan, 250033 China; 9 The II. Medizinische Klinik und Poliklinik der Technischen Universität München, Ismaningerstr. 22, 81675 Munich, Germany

## Abstract

**Background:**

Our previous study have shown that the PSMD11 protein was an important survival factor for cancer cells except for its key role in regulation of assembly and activity of the 26S proteasome. To further investigate the role of PSMD11 in carcinogenesis, we constructed a conditional exon 5 floxed allele of PSMD11 (PSMD11^*flx*^) in mice.

**Results:**

It was found that homozygous PSMD11 ^*flx*/*flx*^ mice showed normal and exhibited a normal life span and fertility, and showed roughly equivalent expression of PSMD11 in various tissues, suggesting that the floxed allele maintained the wild-type function. Cre recombinase could induce efficient knockout of the floxed PSMD11 allele both in vitro and in vivo. Mice with constitutive single allele deletion of PSMD11 derived from intercrossing between PSMD11^*flx/flx*^ and CMV-Cre mice were all viable and fertile, and showed apparent growth retardation, suggesting that PSMD11 played a significant role in the development of mice pre- or postnatally. No whole-body PSMD11 deficient embryos (PSMD11^−/−^) were identified in E7.5–8.5 embryos in uteros, indicating that double allele knockout of PSMD11 leads to early embryonic lethality. To avoid embryonic lethality produced by whole-body PSMD11 deletion, we further developed conditional PSMD11 global knockout mice with genotype *Flp*;*FSF-R26*^*CAG − CreERT2/+*^; *PSMD11* ^*flx*/*flx*^, and demonstrated that PSMD11 could be depleted in a temporal and tissue-specific manner. Meanwhile, it was found that depletion of PSMD11 could induce massive apoptosis in MEFs.

**Conclusions:**

In summary, our data demonstrated that we have successfully generated a conditional knockout allele of PSMD11 in mice, and found that PSMD11 played a key role in early and postnatal development in mice, the PSMD11 ^*flx*/*flx*^ mice will be an invaluable tool to explore the functions of PSMD11 in development and diseases.

**Supplementary Information:**

The online version contains supplementary material available at 10.1186/s12861-020-00233-1.

## Background

In living mammalian cells, it has been estimated that more than 80% of the protein degradation is catalyzed by the 26S multiprotein complex proteasome [1], thus the proteasome has significant role in many of the biological processes, such as apoptosis, cell cycle regulation, antigen presentation and DNA damage repair [2]. The 26S proteasome consists of the 670 kDa proteolytic core particle (CP) or the 20S proteasome, and the 900 kDa regulatory particle (RP), or the 19S complex. The RP is made up of a lid comprising 9 subunits including PSMD11(Proteasome 26 s subunit, non-ATPase, 11), and a base which contains 6 ATPases and several other components [3, 4]. The 19S regulatory subunit is responsible for recognizing and binding the ubiquitin-protein conjugates and transporting the proteins into the CP to be degraded [5, 6].

In recent years, more and more diseases have been found to be associated with the dysfunction of proteasome, such as neurodegenerative diseases, cancer and aging [7, 8]. In order to understand the role of proteasome in these diseases, knockout studies for several subunits of proteasome have been conducted in mice, including Psmc3 and Psmc4 [9], Psmc1 [10], and Psmd4 [11], and found that proteasome play an indispensable role in early-embryonic development of mice and pathogenesis of some of the neurodegenerative diseases [10, 12].

The PSMD11 (Proteasome 26 s subunit, non-ATPase, 11) protein (also known as RPN-6 in *Caenorhabditis elegans*) [13–15] is a component of the lid of the 19S RP, and plays a key role in the regulation of assembly and activity of the 26S proteasome [16, 17]. Additionally, PSMD11 protein was also found to be an important survival factor for cancer cells [18, 19]. Quick degradation of PSMD11 protein has been found to be associated with acute apoptosis in cancer cells, and its knockdown by siRNA can promote acute apoptosis in pancreatic cancer cells [18], suggesting that PSMD11 may be a multi-functional protein, and may be a novel therapeutic target for cancer, whether it can be targeted to treat cancer deserve further investigation.

To further delineate the role of PSMD11 in the process of carcinogenesis, we generated a conditional exon 5 floxed allele of PSMD11 (PSMD11^*flx*^), and demonstrated that Cre recombinase could induce efficient knockout of the floxed PSMD11 allele both in vitro and in vivo in a temporal and tissue-specific manner*.* This novel conditional knockout mice of PSMD11 (PSMD11 ^*flx*/*flx*^) will be very helpful for us to understand the function and mechanism of PSMD11 in physiological and pathological conditions.

## Results

### Construction and identification of a conditional floxed allele of PSMD11 in mice

**Fig. 1:**
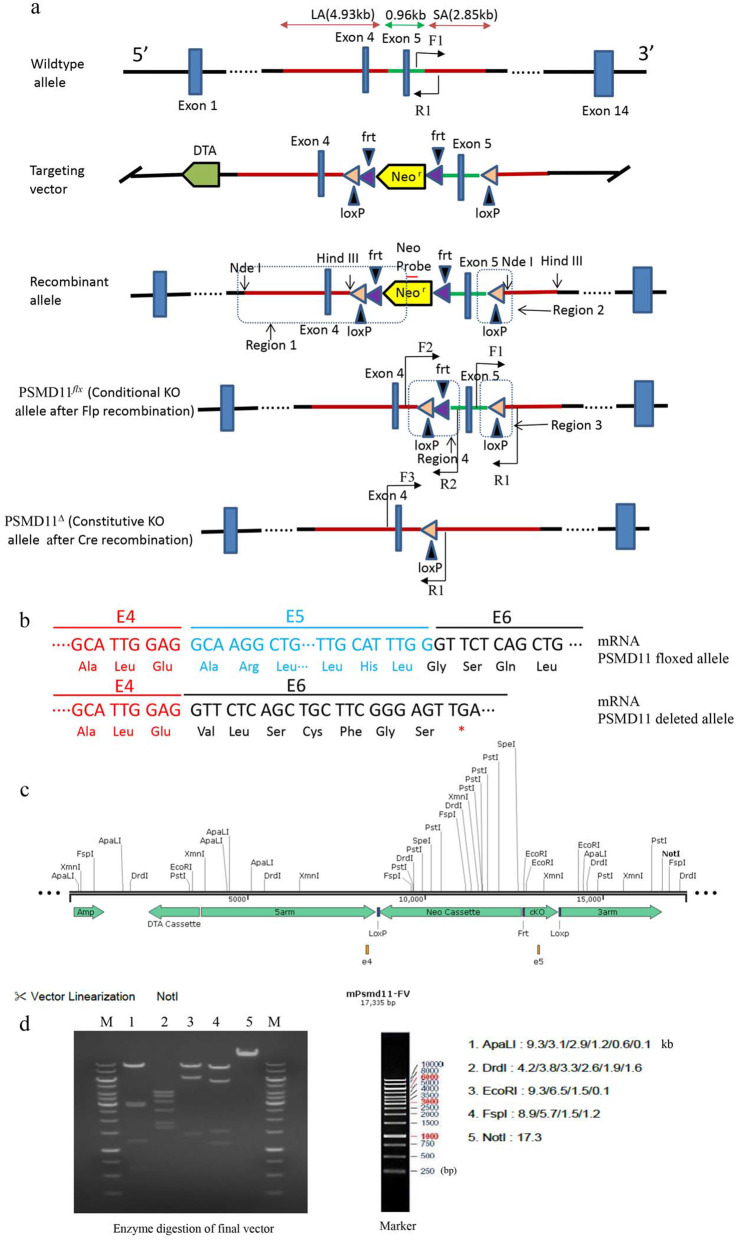
Construction and identification of a Conditional Targeting Vector For PSMD11. **a** Schematic figures of the wild-type allele of PSMD11, the targeting vector, PSMD11^*neo-flx*^ (recombinant allele), PSMD11^*flx*^(conditional floxed allele after Flp recombination) and PSMD11^∆^(constitutive KO allele after Cre recombination) alleles. Blue boxes, Brown triangles and purple triangles represent exons with the exon number indicated, *loxP* sites and *frt* sites separately. The 5′ long homology arm (LA) including exon 4 of the targeting vector is∼4.93 kb, the 3′ short homology arm (SA) is∼2.85 kb. Small Red line denote the location of Neo probe for Southern blot. Arrows show the location of the primers for sequencing and genotyping. The F1 PSMD11 ^*flx-neo/+*^ mice was crossed with the FLPe deleter mice to remove the *Neo* cassette in the germline. PSMD11^*flx/flx*^ mice were mated with CMV-Cre mice to generate PSMD11^∆/+^ mice. **b** Comparison of mRNA transcripts from PSMD11 exon 5 floxed allele or deleted allele to show the introduction of a stop codon (*) after removal of the E5 in the chimeric transcript of E4 and E6. The predicted amino acids are shown below the mRNA labeled in colors. **c** and **d** Restriction endonuclease pattern and analysis of the targeting vector which was digested with restriction endonuclease ApaLI, DrdI, EcoRI, FspI5 or NotI respectively, the DNA fragments was separated by 0.5% gel electrophoresis

**Fig. 2:**
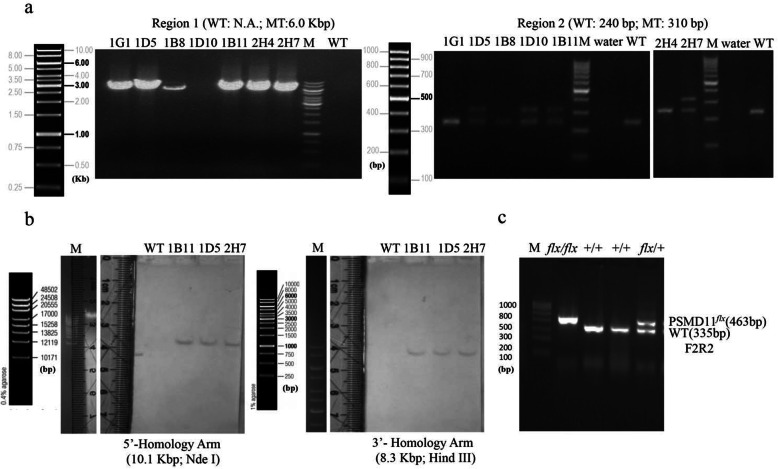
Generation of mice with floxed allele of PSMD11. **a** PCR amplification of region 1 and 2 with primer A1 and A2, or B1 and B2 to identify appropriately targeted embryonic stem cells. Size of the resulting PCR products was 6 kb (MT) for primer A1 and A2, and 310 bp (MT,) and 240 bp (WT) for primer B1 and B2 respectively. **b** reconfirmation of targeted embryonic stem cell clones by southern blotting. Nde I and Hind III digested DNA from three individual clones that had passed all controls was electrophoretically separated on a 0.5% agarose gel, after transfer to a nylon membrane, a probe targeted against the Neo cassette was hybridized with the digested DNA, which showed two bands at 10.1 kb, and 8.3 kb respectively. **c** Genotyping of mice with genomic DNA from tail biopsies. Primers F2 and R2 were used to distinguish the floxed allele (PSMD11^*flx*^, 463 bp) from the wild-type allele (WT, 335 bp)

The murine PSMD11 gene, about 44.62 kb in length, is situated on the reverse strand of chromosome 11, and consists of 14 exons in which the ATG start codon is in exon 1 and TAG stop codon in exon 13 (Fig. 1a). To construct a functional conditional knockout allele of PSMD11 in mice, we selected exon 5 as conditional knockout target by insertion of flanking *loxP* sequences for its 100% sequence homology with the human PSMD11 exon 5, which indicated a highly conserved function for this exon. In addition, deletion of exon 5 can lead to a formation of a stop codon in the chimeric transcript of exon 4 and 6 (Fig. 1b). To construct the floxed allele of PSMD11 (PSMD11^*flx*^) in mice, two relevant genomic DNA fragments in the murine BAC library was selected; a *loxP* sequence and a frt-flanked Neo expression cassette for positive selection were inserted 220 bp downstream from exon 4, another *loxP* sequence was inserted 549 bp downstream from exon 5. After confirmed by restriction analysis with restriction endonuclease ApaLI, DrdI, EcoRI, FspI5 or NotI (Fig. 1c and d) and sequencing (Additional file 1: Figure S1) with S_14_ primers (Table 1), the linearized targeting vector was eletroporated into C57BL/6 embryonic stem cells, then G418 treatment, PCR (Fig. 2a) and Southern blotting (Fig. 2b) were performed to screen the correctly recombined ES cell colonies. Three correctly recombined clones (1B11, 1D5, 2H7) were injected into C57BL/6 blastocysts. Germline transmission was obtained in 3/3 clones. Then, the *Neo* cassette in the germline was removed through intercrossing F1 PSMD11 ^*flx-neo/+*^ mice with the FLPe deleter mice [20]. Then the resulted PSMD11^*flx*/+^ offspring were interbred to obtain homozygous PSMD11 floxed (PSMD11 ^*flx*/*flx*^) mice. The homozygous PSMD11 ^*flx*/*flx*^ mice can be detected at the expected Mendelian ratio by Genotyping (Fig. 2c).

**Table 1:** Oligonucleotides used in this study

Name	Sequence	Purpose
Region 1-F Region 1-R Region 2-F Region 2-R Neo-F Neo-R Region 3-F Region 3-R Region 4-F Region 4-R CRE-F CRE-R F1 R1 F2 R2 F3 S_1_ S_2_ S_3_ S_4_ GAPDH-F GAPDH-R PSMD11-F PSMD11-R Flp-F Flp-R Probe for neo	5′-tccatgtctgggctctctactgtttc-3′ 5′-gctgaccgcttcctcgtgcttta-3′ 5′-tatgtctgtgagggtgttggatac-3′ 5′-cagggatccaacatttctga-3′ 5′-tcatctcaccttgctcctgc-3′ 5′-aaggcgatagaaggcgatgc-3′ 5′-tatgtctgtgagggtgttggatac-3′ 5′-cagggatccaacatttctga-3′ 5′-tagatagtgcttccagttatgggag-3′ 5′-gactgttacctacaccatactgctg-3′ 5′-atttgcctgcattaccggtc-3′ 5′-atcaacgttttcttttcgg-3′ 5′-tatgtctgtgagggtgttggatac-3′ 5′-cagggatccaacatttctga-3′ 5′-tagatagtgcttccagttatgggag-3′ 5′-gactgttacctacaccatactgctg-3′ 5′-gttagttggaagaaggtaagggc-3′ 5′-attcgccaatgacaagacgc − 3′ 5′-aacttatagggatttgcctgctacttt-3′ 5′-aaaagaactctagggttgc-3′ 5′-aatctgcctgcctctgcctcccatgtgc-3′ 5′-aaaagggtcatcatctccg-3′ 5′-agtcttctgagtggcagtgat-3′ 5′-agagcagagcatccttgaact-3′ 5′-tcggcagattactcagagcat-3′ 5′-cactgatattgtaagtagtttgc-3′ 5′-ctagtgcgaagtagtgatcagg-3′ 5′-aaggcgatagaaggcgatgcgctgcgaatcgggagcggcgataccgtaaagc acgaggaagcggtcagcccattcgccgccaagctcttcagcaatatcacgggtagc caacgctatgtcctgatagcggtccgccacacccagccggccacagtcgatgaatccagaaaagcggccattttccaccatgatattcggcaagcaggcatcgccatgggtcacgacgagatcatcgccgtcgggcatgcgcgccttgagcctggcgaacagttcggctggcgcgagcccctgatgctcttcgtccagatcatcctgatcgacaagaccggcttccatccgagtacgtgctcgctcgatgcgatgtttcgcttggtggtcgaatgggcaggtagccggatcaagcGtatgcagccgccgcattgcatcagccatgatggatactttctcggcaggagcaaggtgagatga-3’	Gene targeting Gene targeting Gene targeting Gene targeting Gene targeting Gene targeting Gene targeting Gene targeting Gene targeting Gene targeting Gene targeting Gene targeting Gene targeting Gene targeting Gene targeting Gene targeting Gene targeting Sequencing Sequencing Sequencing Sequencing qRT-PCR qRT-PCR qRT-PCR qRT-PCR Gene targeting Gene targeting Southern blot

### Floxed PSMD11 gene could be deleted efficiently in vitro with Cre-mediated gene recombination

**Fig. 3:**
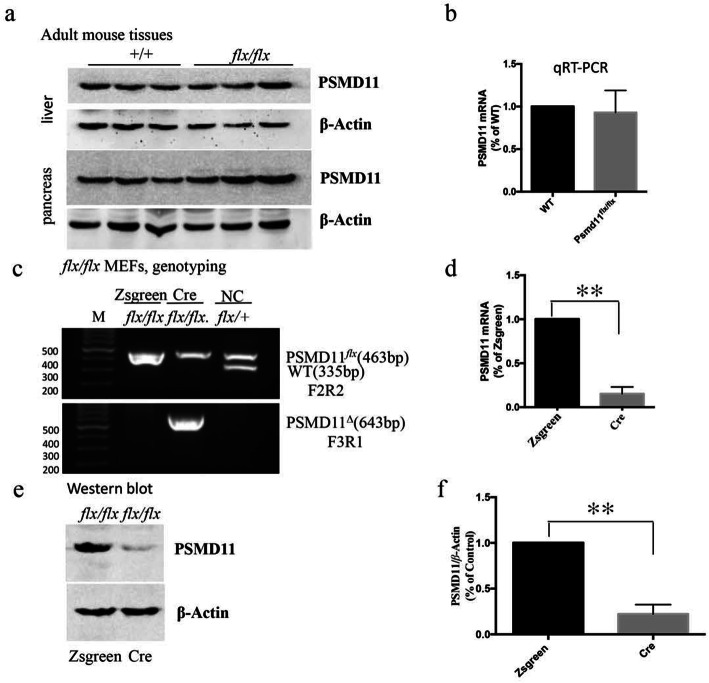
Floxed PSMD11 allele could be deleted efficiently in vitro with Cre-mediated gene recombination**. a** No difference of PSMD11 expression could be found in liver and pancreas between adult WT and PSMD11^*flx/flx*^ mice by western blot. *n* = 3 each group. **b** qRT-PCR to detect PSMD11 mRNA expression in RNA from adult WT and PSMD11 ^*flx/flx*^ mouse liver and pancreas tissues, *n* = 3 each group. **c** PCR analysis of DNA from AAV (expressing ZsGreen or Cre) infected PSMD11 ^*flx/flx*^ MEFs and a nonrecombined PSMD11 ^*flx/+*^ mouse without Cre expression (NC), primer F3 and R1 will produce 643 bp bands in PSMD11 exon 5 deleted alleles, primers F2 and R2 will produce 463 bp and 335 bp bands in PSMD11 exon 5 floxed or WT alleles respectively. **d** Quantification of PSMD11 mRNA by real-time RT-PCR on total RNA extracted from AAV (expressing ZsGreen or Cre) infected PSMD11 ^*flx/flx*^ MEFs. **e** Western blot to show nearly complete depletion of PSMD11 protein in Cre-infected PSMD11 ^*flx/flx*^ MEFs. **f** Quantification of PSMD11 protein expression in **e** which was normalized to β-Actin and expressed as percent of the controls. The mean ± SEM of two independent experiments was shown. **P < 0.01, *P < 0.05

Homozygous PSMD11 ^*flx*/*flx*^ mice appeared normal and fertile, and had a normal life span. Roughly equivalent endogenous expression of PSMD11 could be detected in various tissues such as liver and pancreas at both protein and mRNA level in both PSMD11 ^*flx*/*flx*^ mice and age matched WT mice (Fig. 3a and b). To demonstrate whether the floxed allele of PSMD11 could be knocked out by Cre in vitro, we isolated MEFs (mouse embryonic fibroblasts) from E14.5 embryos of intercrosses between PSMD11 ^*flx*/*flx*^ mice and infected with Adeno-associated virus (AAV) expressing either ZsGreen or Cre. Genomic PCR genotyping and sequencing confirmed the recombination of the floxed PSMD11allele after 5 days following infection (Fig. 3c and Supporting Information 1). Near complete loss of mRNA and protein expression of PSMD11 could also be found by qRT-PCR and Western blot respectively (Fig. 3d-f). The loss of PSMD11 mRNA indicated that deletion of exon 5 could probably lead to premature transcription termination or mRNA decay due to the premature stop codon [21].

### Constitutive ablation of PSMD11 leads to early embryonic lethality (PSMD11^−/−^) and growth retardation (PSMD11^+/−^) in mice

**Fig. 4:**
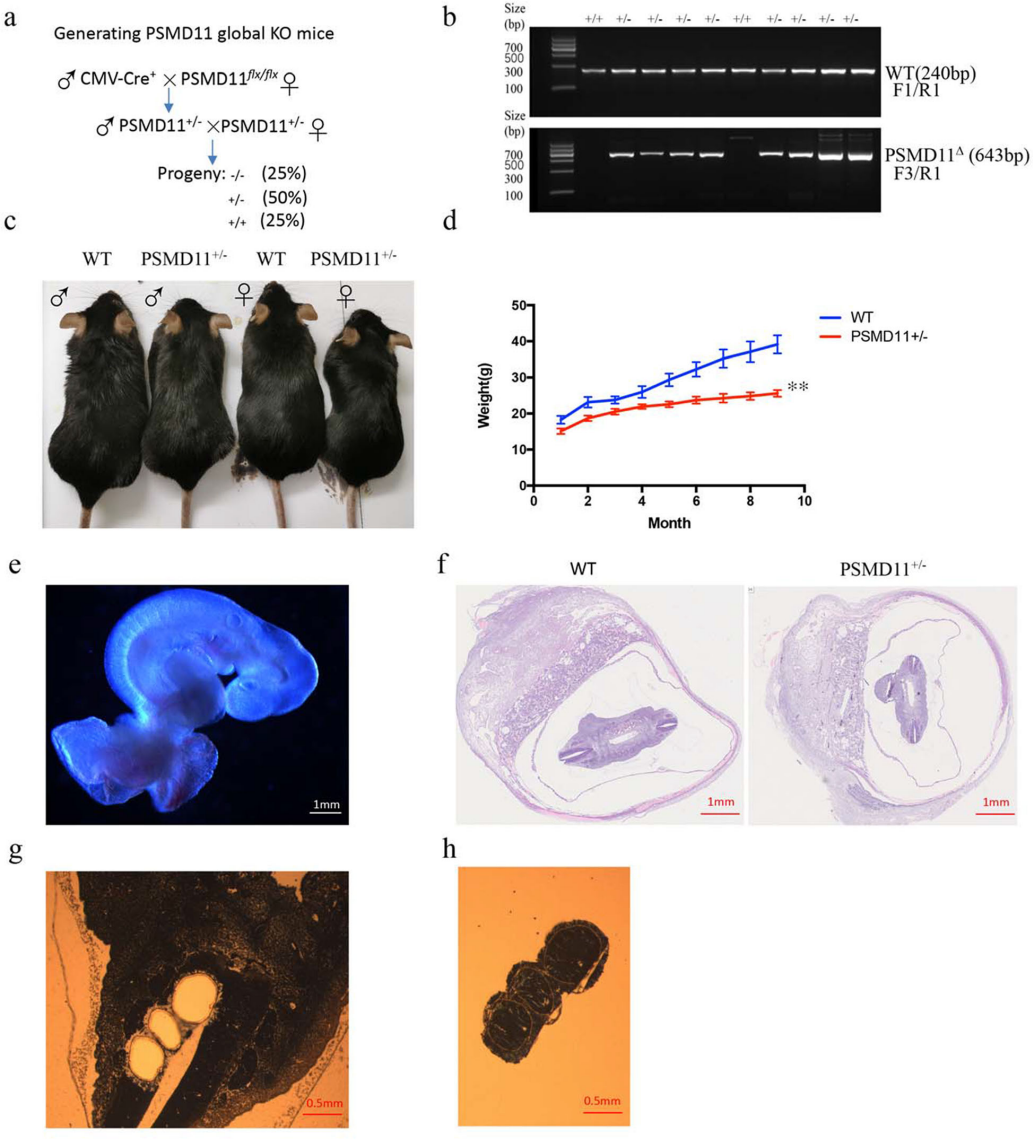
Constitutive ablation of PSMD11 leads to early embryonic lethality (PSMD11^−/−^) and growth retardation (PSMD11^+/−^) in mice. **a** Methods to generate PSMD11 constitutive KO mice. **b** Genotyping of E7.5–8.5 embryos from intercrossing between PSMD11^+/−^ heterozygous mice. The 643 bp band indicate the exon 5 deleted allele of PSMD11 (PSMD11^∆^). **c** Representative Smaller body size of PSMD11^+/−^ mice compared with WT littermates. **d** Growth curve of PSMD11^+/−^ mice (*n* = 9) and age- and sex-matched male and female WT littermates (*n* = 9), **P < 0.01, *P < 0.05. **e** Representative image of E7.5–8.5 embryos of mice under stereomicroscope. **f** Representative HE images of E7.5–8.5 WT and PSMD11^+/−^ embryos of mice. **g** and **h** Representative images of Arcturus Histogene™ staining and Laser Capture Microdissected tissues of E7.5–8.5 embryos of mice

To verify if the floxed allele of PSMD11 could be knocked out by Cre in vivo, the PSMD11^*flx/flx*^ mice were crossed with cytomegalovirus (CMV)-Cre mice (Fig. 4a) to induce constitutive deletion of the floxed allele of PSMD11 in whole body [22]. The resultant heterozygous male and female PSMD11^+/−^ mice could be identified at the expected Mendelian ratio, and they appeared normal except that they show apparent growth retardation compared with age- and sex-matched male and female WT mice (Fig. 4b-d), which was consistent with the findings that proteasome inhibition with proteasome inhibitor bortezomib could lead to severe growth retardation in mice through induction of apoptosis of stem-like and proliferative chondrocytes in the growth plate [23].

To further obtain PSMD11 double allele knocked out mice (PSMD11^−/−^), the heterozygous PSMD11^+/−^ male and female mice were intercrossed. In the viable progeny, no PSMD11^−/−^ mice were identified after birth (0 of 89 mice), indicating that double allele deletion of PSMD11 leads to embryonic lethality. To identify the time of embryo death, embryos of E7.5–8.5 in utero were collected (Fig. 4e, f), global KO embryos were still not identified by Laser-capture microdissection (Fig. 4g, h) and PCR genotyping. The heterozygous PSMD11^+/−^ and WT male and female mice could be identified at the ratio of 77.08% (37/48) and 22.92% (11/48) respectively, further indicating that PSMD11 was essential for the survival of early embryo, or even the zygotes, although it still needs to be demonstrated. The male and female PSMD11^+/−^ mice were all fertile when backcrossed to C57BL/6 J mice, and the heterozygous PSMD11^+/−^ offspring mice could be collected at the expected Mendelian ratio.

### PSMD11 could be conditionally depleted in vitro and in vivo and could induce massive apoptosis in mouse embryonic fibroblasts (MEFs)

**Fig. 5:**
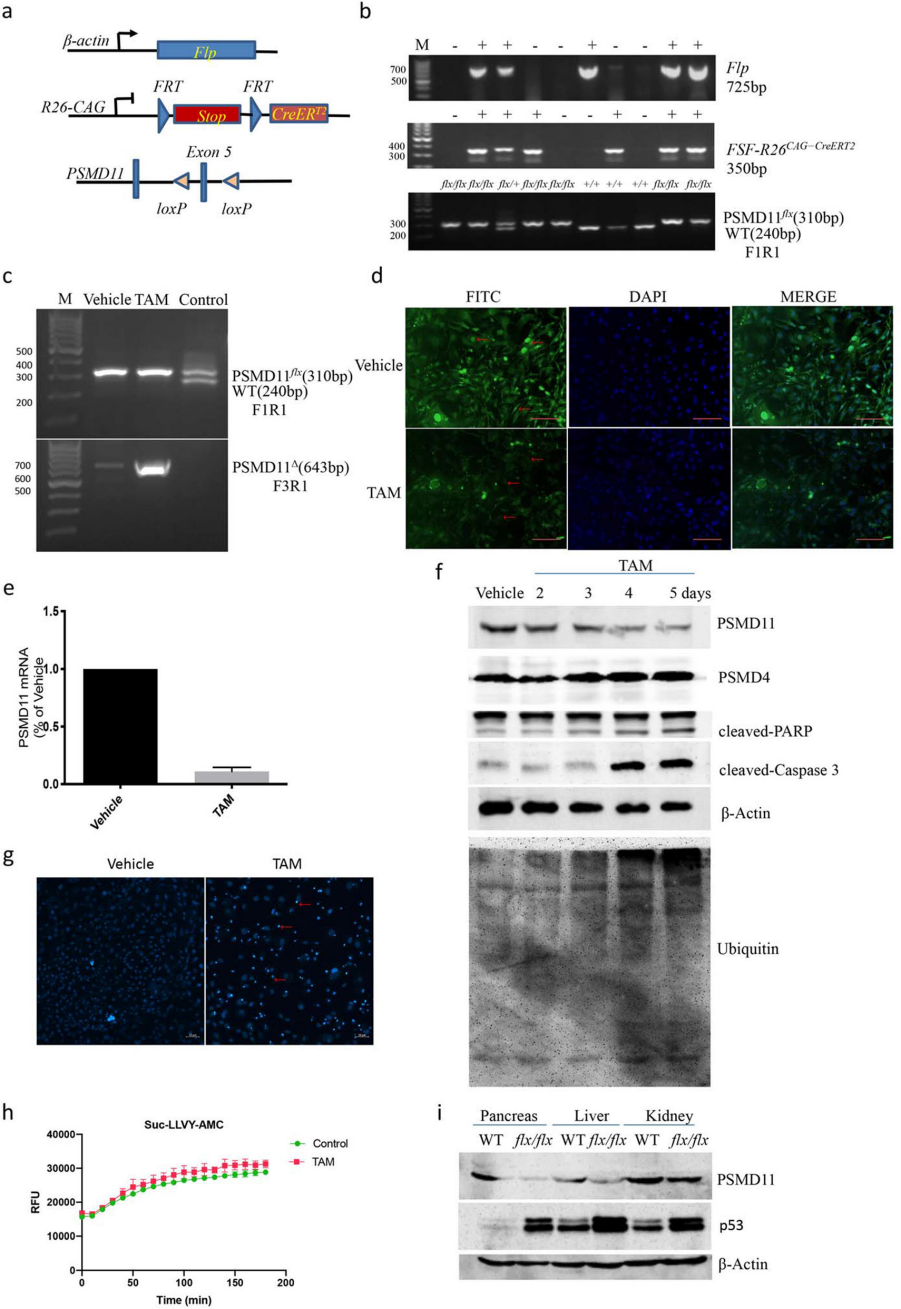
PSMD11 could be conditionally depleted in vitro and in vivo and could induce massive apoptosis in MEFs. **a** Genetic strategy to knockout PSMD11 by tamoxifen-mediated CreER^T2^ activation. **b** Genotyping of DNA from E14.5 embryos from intercrossing between *Flp;FSF-R26*^*CAG − CreERT2/+*^;*PSMD11* ^*flx*/*+*^ mice. Sizes of WT and mutant PCR bands are indicated. **c-e** MEFs were isolated by a standard protocol from E14.5 embryos with the genotype of *Flp;FSF-R26*^*CAG − CreERT2/+*^;PSMD11^*flx/flx*^, then the cells were treated for 5 days with 0.5uM 4-hydroxytamoxifen or vehicle (ethanol) to induce knockout of PSMD11, then Genotyping (**c**), immunofluorescence (**d**) and qRT-PCR (**e**) were performed to detect the change of expression of PSMD11. Control DNA was from a nonrecombined PSMD11 ^*flx/+*^ mouse without Cre expression, nuclei are counterstained with DAPI (blue) in immunofluorescence staining. Scale bars, 50 μm. **f** MEFs were treated with 0.5uM 4-hydroxytamoxifen or vehicle (ethanol) for the indicated times, then Western blotting was performed with antibodies directed against PSMD11, PSMD4, PARP, cleaved-Caspase 3, Ubiquitin, β-actin was used as loading control. **g** After treated with 0.5uM 4-hydroxytamoxifen or vehicle (ethanol) for 5 days, MEFs were stained with 1 μg/mL Hoechst 33342 for 20 min and imaged by confocal laser-scanning microscopy. Arrows denote apoptotic cells. **h** The chymotrypsin-like activity of proteasome was tested in 30 μg whole-cell extracts from vehicle or tamoxifen treated fibroblasts for 4 days with 0.1 mM Suc-LLVY-AMC peptide at 37 °C for 180 min in quadruplicates on a 96-well plate, Y-axis indicated the relative fluorescence units (RFU) reflecting the AMC cleavage from the peptide. Bars showed the mean ± SD. **i** The FCP ^*flx*/*flx*^ and WT mice were fed with tamoxifen-containing food (400 mg/kg) or vehicle for 3 days, then the pancreas, liver and kidney were collected to detect the expression of PSMD11 and P53 with β-actin as loading control with Western blotting

To avoid embryonic lethality caused by whole-body PSMD11 deletion, we further developed conditional PSMD11 global knockout mice with genotype *Flp*;*FSF-R26*^*CAG − CreERT2/+*^; *PSMD11* ^*flx*/*flx*^ (FCP ^*flx*/*flx*^) by crossing the human beta-actin FLPe deleter strain of mice with the *FSF-R26*^*CAG − CreERT2/+*^ mice [24] and homozygous PSMD11 ^*flx*/*flx*^ mice, in which *Flp* induced deletion of the *FRT*-stop-*FRT* (*FSF*) cassette could activate the latent tamoxifen-inducible allele *CreER*^*T2*^ (Fig. 5a and b). The FCP ^*flx*/*flx*^ mice appeared normal and fertile. For validating PSMD11 depletion, MEFs were isolated from mouse E14.5 embryos with genotype *Flp*;*FSF-R26*^*CAG − CreERT2/+*^;*PSMD11* ^*flx*/*flx*^, and treated with 0.5uM 4-hydroxytamoxifen or vehicle (ethanol) for 5 days to induce deletion of the floxed allele of PSMD11. As shown in Fig. 5c, the deleted allele of PSMD11 (PSMD11^Δ^, 643 bp) could be detected in the 4-hydroxytamoxifen treated MEFs, loss of protein expression of PSMD11 in the cytoplasm and nucleus of the majority of MEFs could be found by immunofluorescence staining (red arrows in Fig. 5d), qRT-PCR further confirmed that PSMD11 transcripts (Fig. 5e) could be depleted significantly.

For it had been shown that knockdown of PSMD11 by siRNA could promote acute apoptosis in pancreatic cancer cells [18], we also detected if depletion of PSMD11 could induce apoptosis in MEFs. As shown in Fig. 5f and g, treatment with 0.5uM 4-hydroxytamoxifen for 5 days could gradually deplete the protein expression of PSMD11. The intracellular level of ubiquitinated proteins was found to be increased following the treatment with 4-hydroxytamoxifen, suggesting that the proteasomal function was compromised. No change of expression of PSMD4 was found, another component of the he 19S RP of the 26S proteasome. The apoptosis-specific cleaved-PARP and cleaved caspase 3 increased with the depletion of PSMD11, and a great deal of cells with nuclear condensation and fragmentation could be found by Hoechst 33342 staining, demonstrating that depletion of PSMD11 could induce apoptosis in MEFs.

To further characterize the effect of PSMD11 depletion on the functionality of the 26S proteasome, the chymotrypsin like activity of both 20S and 26S complexes was monitored for 180 min by measuring directly the degradation rate of the peptide Suc-LLVY-AMC in whole-cell extracts from vehicle and tamoxifen treated fibroblasts for 4 days, no significant differences was found (Fig. 5h), suggesting that PSMD11 depletion had no obvious effect on the peptide hydrolysis in the 20S proteolytic core, at least in this time point.

To detect whether PSMD11 could be depleted in a tissue-specific manner in vivo, the FCP ^*flx*/*flx*^ mice were fed with tamoxifen-containing food (400 mg/kg) for 3 days, then we detected the expression of PSMD11 in pancreas, liver and kidney. As shown in Fig. 5i, tamoxifen could induce efficient PSMD11 depletion in pancreas and liver, and partial depletion in kidney, suggesting that the conditional knockout system also works in a tissue-specific manner in vivo. Meanwhile, it was found that the P53 protein was up-regulated following down-regulation of PSMD11, which is consistent with the findings that proteasome inhibition could up-regulates p53 [23]. The biologic effect of PSMD11 global knockout in adult mice is under investigation.

## Discussion

To further delineate the function of PSMD11 in vivo, we generated a conditional exon 5 floxed allele of PSMD11 (PSMD11^*flx*^) in mice. It was found that homozygous PSMD11 ^*flx*/*flx*^ mice were normal and fertile, and showed normal expression of PSMD11 in various tissues, suggesting that the floxed allele maintained the wild-type function. Cre could induce efficient knockout of the floxed allele of PSMD11 both in vitro and in vivo. Mice with constitutive single allele deletion of PSMD11 obtained through intercrossing between PSMD11^*flx/flx*^ and CMV-Cre mice showed apparent growth retardation compared with the age-and sex-matched WT mice, suggesting that PSMD11 have important role in the development of mice pre- or postnatally. No whole-body PSMD11 deficient E7.5–8.5 embryos (PSMD11^−/−^) were identified in uteros, indicating that double allele knockout of PSMD11 led to early embryonic lethality. To avoid embryonic lethality elicited by whole-body PSMD11 deletion, we further developed conditional PSMD11 global knockout mice with genotype *Flp*;*FSF-R26*^*CAG − CreERT2/+*^;*PSMD11* ^*flx*/*flx*^, and demonstrated that PSMD11 could be depleted in a temporal and tissue-specific manner. Meanwhile, it was found that depletion of PSMD11 could induce massive apoptosis in MEFs, further indicating that PSMD11 was a significant survival factor for cells, whether it could be targeted to treat cancer deserve further investigation.

Proteasomes have important role in many of the biologic processes such as survival, DNA repair and proliferation of malignant cells, and RAS mutant cancer cells have been shown to be selectively addicted to proteasome activity [25–27]. However, proteasome inhibitors (PIs) are only effective in hematologic malignancies including multiple myeloma and mantle cell lymphoma [28]. For solid tumors, PIs only showed marginal effect [29, 30], the reason was not clear. For the proteasome inhibitors current in use all work primarily at the chymotrypsin-like site on β5 subunit of the 20S catalytic core of proteasome [31], one of the reasons may be that the chymotrypsin-like site on β5 is not an appropriate therapeutic target for solid cancers, thus whether other components of proteasome can be targeted to treat cancer deserve further investigation.

PSMD11 is a component of the lid of the 19S RP, it can hold the 20S CP and 19S RP together [14] like a molecular clamp through protein-protein interactions with Rpt-6 of the ATPase ring of the 19S RP and the a-2 subunit of the 20S CP [32]. In this study, it was found that deletion of PSMD11 could lead to increased intracellular levels of ubiquitinated proteins, but had no obvious effect on the peptide hydrolysis activity of the 20S proteolytic core, further confirmed that PSMD11 played an essential role in the RP to recognize, unfold and translocate the ubiquitinated-proteins into the 20S proteolytic cavity of the proteasome [33]. For depletion of PSMD11 could induce massive apoptosis in MEFs, it also indicated that the 20S proteolytic CP alone was unable to maintain survival of the MEFs.

PSMD11 constitutively deficient mice have never been reported previously so far. In this study, it was found that PSMD11 single allele deleted mice (PSMD11^+/−^) showed evident growth retardation, whereas double allele deleted mice (PSMD11^−/−^) showed early embryonic lethality, indicating that PSMD11 had important role in the development of mice pre- or postnatally. Meanwhile, it was found that depletion of PSMD11 led to up-regulation of P53 in the conditional knockout mice of PSMD11, suggesting that regulation of the expression level of some of the critical proteins might be one of the possible mechanisms for regulation of development by PSMD11 or proteasome. In the following study, we will try to identify these critical proteins with proteomics.

One of the limitations of our study is that we did not characterize the time and reason of embryo death of PSMD11 double allele deleted mice. Taking into account the findings that hESCs (human embryonic stem cells) were highly sensitive to proteasome inhibition [16], and depletion of PSMD11 could induce massive apoptosis in MEFs, we speculate that the death time of embryo may be at the zygote stage, further study with our conditional PSMD11 global knockout mice will be helpful to resolve this question.

## Conclusions

In summary, our data demonstrated that we have successfully generated a conditional knockout allele of PSMD11 in mice, and found that PSMD11 played a key role in early and postnatal development in mice. This novel conditional knockout mice of PSMD11 will be very helpful for us to understand the function and mechanism of PSMD11 in physiological and pathological conditions.

## Methods

### Materials

Cell culture reagents were all purchased from Invitrogen (Groningen, the Netherlands). Primers were synthesized by Sangon Biotech (Shanghai, China). Restriction endonucleases were got from New England BioLabs (Mannheim, Germany). Transformation and plasmid amplification was performed with TOP10 and Stbl3 *E. coli* strains (Invitrogen, Groningen, the Netherlands). Tamoxifen Citrate (S1972) and 4-Hydroxytamoxifen (S7827) were purchased from Selleck (Shanghai, China).

### Mouse strains

CMV-Cre mice (Jackson lab, stock#: 006054) (ref. [15]), *FSF-R26*^*CAG − CreERT2/+*^ mice (ref. [17]), the human beta-actin FLPe deleter strain of mice (B6;SJL-Tg (ACTFLPe)9205Dym/J, the Jackson Laboratory, Stock Number: 003800) and *R26*^*CAG − CreERT2/+*^ mice [24] have been described previously [20]. The mice were all on a mixed *C57Bl/6*;*129S6/SvEv* genetic background. All mice were kept at a SPF environment, no mice were excluded from analyses. Experiments with mice were performed with protocols approved by the Shandong University Institutional Animal Care and Use Committee, complying with the rules of Regulations for the Administration of Affairs Concerning Experimental Animals (Approved by the State Council of China). Mice after the study were all euthanized by cervical dislocation.

### Targeting vector construction for PSMD11

The floxed allele of PSMD11 was generated with traditional gene targeting methods in collaboration with Cyagen Biosciences (Guangzhou, China). The targeting vector was constructed with positive BAC clone RP23-307G3 and RP23-276 J12 from the C57BL/6 J library. In the targeting vector, the long homology arm (LA) including exon4(4.93 kb), the short homology arm (SA, 2.85 kb), and the conditional knockout region (cKO) including exon 5 were generated by PCR with high fidelity Taq DNA polymerase, and then sequentially integrated into the targeting vector with recombination sites and selection markers. A loxP/frt Neo cassette including exon 5 was inserted 220 bp downstream from exon 4, two frt sites flanked the Neo cassette for later Flp (Flippase) recombinase excision, it also contained the promoter of the mouse phosphoglucokinase gene and a synthetic polyadenylation sequence. The cassette was flanked by two loxP sites, one outside the frt sites, one outside the exon 5. The targeting vector was 17.3 kb totally including the vector backbone and Neo cassette. Restriction analysis with restriction endonuclease ApaLI, DrdI, EcoRI, FspI5 and NotI was performed to confirm the structure of the targeting vector respectively. Sequence of connection region between vector backbone and homology arm or the conditional knockout region (cKO) was confirmed by sequencing with S_1–4_ primers (Table 1).

### Targeted embryonic stem (ES) cells

The linearized targeting vector (10 μg) by NotI was electroporated into C57BL/6 ES cells, then the ES cells were selected with G418, total 175 drug-resistant clones were obtained. After expansion of the surviving clones, genotyping by PCR was applied to identify correctly recombinant clones with primer A1 and A2 for region 1, B1 and B2 for region 2 (Table 1). Size of the PCR bands were 6 kb (MT) for primers A1 and A2, and 310 bp (MT) and 240 bp (WT) for primers B1 and B2 respectively. Three ES clones which passed all the controls were further reconfirmed by Southern bloting. DNA digested with Nde I and Hind III was separated on a 0.5% agarose gel, then transferred onto a PVDF membrane, a probe targeting the Neo cassette was hybridized with the digested DNA, which resulted in two bands at 10.1 kb, and 8.3 kb respectively. DNA from wild-type C57Bl/6 (B6) mice were used as controls.

### Generation of floxed allele of PSMD11 in mice

Three correctly targeted ES clones were used to microinject into C57BL/6 blastocysts. Germline transmission was gained in 3/3 clones harboring the targeted allele. Then, the *Neo* cassette in the germline was removed by crossing the F1 PSMD11 ^*flx-neo/+*^ mice with the FLPe deleter mice. Then the resulted PSMD11^*flx*/+^ offspring were interbred to obtain homozygous PSMD11 floxed (PSMD11 ^*flx*/*flx*^) mice. PCR using F1, R1 and F2, R2 primers (Table 1) was used to genotype mice.

### Generation of PSMD11 constitutively knocked out (KO) mice

Intercrossing between homozygous PSMD11 ^*flx*/*flx*^ mice and CMV-Cre mice was performed to generate the constitutively knocked out (KO) mice of PSMD11. The CMV-Cre mice express Cre ubiquitously, and can delete floxed gene in all the tissues, including germ cells. The obtained heterozygous mice with single allele deletion (PSMD11^*+/−*^) were then intercrossed to generate PSMD11 global knockout mice (PSMD11^*−/−*^). The genomic sequence of the PSMD11^*+/−*^ mice after deletion of exon 5 was confirmed by sequencing with F3 and R1 primers (Table 1).

### DNA extraction, genotyping and sanger sequencing

One-tube General Sample DNAup kit for PCR was used to extract DNA following the manufacturer’s protocol (Sangon Biotech, Shanghai, China). PCR genotyping was performed using the primers in Table 1. PCR bands were separated by 1% agarose gel electrophoresis or purified by an agarose gel extraction kit (TransGen Biotech, Beijing, China) for Sanger sequencing (Sangon Biotech, Shanghai, China).

### Protein extraction and Western Bloting

Protein was extracted from tissues or cells on ice using RIPA with 1% proteinase inhibitor cocktail (Sigma-Aldrich, Shanghai, China) and 1% phenylmethane sulfonyl fluoride. Protein concentration was determined with a BCA assay kit. 30-50 μg of total protein was resolved with a 10% SDS-PAGE gel, then transferred to nitrocellulose membranes (Millipore). The membranes was blocked with 5% nonfat milk in TBS and 0.1% Tween20, the anti-PSMD11 (14786–1-AP) and anti-PSMD4 antibody (14899–1-AP) from Proteintech Group, anti-PARP (#9542), anti-cleaved-caspase 3 (#9664) and anti-k48-linkage specific polyubiquitin(#12805) antibody from Cell Signaling Technology, anti-p53 antibody (sc-55,476) from Santa Cruz Biotechnology, mouse anti-β-actin and HRP conjugated secondary anti-mouse and anti-rabbit antibodies (Zhongshan Goldenbridge Biotechnology Co., LTD) were used to probe corresponding proteins. Enhanced chemiluminescence (Millipore) was used to visualize the proteins. Images were digitalized with FluorChem Q (Proteinsimple, Santa Clara, CA, USA) at 300 dpi resolution.

### Generation mouse embryonic fibroblasts (MEFs) and knockout of the floxed allele of PSMD11 with Cre

MEFs were isolated from E14.5 mouse embryos by standard protocol. After three passages, MEFs were infected with adeno-associated virus expressing ZsGreen or Cre (Takara, Shanghai, China). Adeno-associated virus (AAV) was prepared by co-transfect 293 T cells with three pAAV vectors, pAAV-CRE, pRC vector, and pHelper vector, with Xfect Transfection Reagent (Takara, Shanghai, China). Purification was accomplished from clarified HEK293 cell lysates by AAV Extraction Solution (Takara, Shanghai, China). Virus titer was determined by measuring the expression of the fluorescent protein ZsGreen. To activate the latent tamoxifen-inducible allele (*CreER*^*T2*^), MEFs with genotype *of Flp*;*FSF-R26*^*CAG − CreERT2/+*^;*PSMD11* ^*flx*/*flx*^ were treated with 0.5uM 4-hydroxytamoxifen or vehicle (ethanol) for 5 days to induce deletion of *loxP*-flanked sequences in vitro. All cell lines were authenticated by genotyping and tested for mycoplasma contamination.

### Real-time RT-PCR for PSMD11 mRNA

Real-time RT-PCR was carried out as described previously [19]. Briefly, RNeasy kit (Qiagen) was used to extract total RNA, 5 μg total RNA was used to synthesize cDNA in the presence of M-MLV reverse transcriptase (100 units, Invitrogen), RNase inhibitor (40 units, Invitrogen) and Oligo (dT)15 primer. Real-time RT-PCR was performed in triplicate with SYBR Green Master Mix (Applied Biosystems) using the QuantStudio 3 (Appliedbiosystems by Thermo Fisher Scientific, Shanghai, China). The 2^−ΔΔCt^ method was used to calculate relative expression of PSMD11 with GAPDH as internal control. The primers for PSMD11 and GAPDH were listed in Table 1.

### Laser-capture microdissection and DNA extraction

Formalin-fixed, paraffin-embedded tissue sections (8 μm thick) of mouse E7.5–8.5 embryos were dewaxed, stained with Histogene™ staining solution (Applied biosystems by Thermo Fisher Scientific, Lithuania) and microdissected using an Arcturus XT laser-capture microdissection system (Appliedbiosystems by Thermo Fisher Scientific, Shanghai, China). The Rapid Animal Genomic DNA Isolation Kit (Sangon Biotech, Shanghai, China) was used to isolated DNA from the microdissected cells.

### Immunofluorescence staining

MEFs were fixed in 4% buffered formalin for 20 min, after washing twice with PBS, it was blocked for 1 h PBS containing 3% (w/v) bovine serum albumin (BSA), 1% (w/v) Saponin and 1% (v/v) Triton X-100, then incubated with rabbit anti-PSMD11 antibody (1:100, 14,786–1-AP, Proteintech Group, Rosemont, USA) for 24 h at 4 °C in the dark followed by FITC conjugated goat anti-rabbit antibodies (#0311, Zhongshan Goldenbridge Biotechnology Co., LTD) for 1 h at room temperature. Nuclei were counterstained with DAPI (1:1000, Invitrogen) for 5 min at room temperature. After three rinses in PBS, slides were mounted with antifading Mounting Medium (Solarbio, Beijing, China) and imaged with fluorescent microscopy with appropriate excitation wavelengths.

### Apoptosis detection with Hoechst 33342 staining and confocal laser-scanning microscopy

MEFs were treated with 0.5uM 4-hydroxytamoxifen or vehicle (ethanol) for 5 days to induce deletion of the floxed allele of PSMD11, after that, the cells were incubated with 1 μg/mL Hoechst 33342 in culture media for 20 min, then photos with a frame size of 1024 × 1024 pixels were taken with Zeiss LSM 800 with airyscan technology (Carl Zeiss, Jena, Germany) with a × 10/0.45 and a × 20/0.8 Plan-APOCHROMAT objective. Images were collected and processed with ZEN 2009 software.

### Detection of proteasome activity in crude protein extracts

The chymotrypsin-like activity of proteasome was detected with 0.1 mM Suc-LLVY-AMC peptide (HY-P1002, MedChemExpress LLC, Shanghai, China) in 100 μl ATP/DTT lysis buffer (Tris-Hcl, pH 7.8, 0.5 mM DTT, 5 mM ATP, Mgcl_2_ 5 mM) at 37 °C in 30 μg whole-cell extracts from control and tamoxifen treated fibroblasts for 4 days. The fluorescence of released aminomethylcoumarin was monitored with a plate reader at an excitation wavelength of 360 nm and emission wavelength of 460 nm over a period of 180 min.

### Tamoxifen induction deletion of PSMD11 in mice

Tamoxifen-containing food (400 mg/kg) was fed to mice for 3 days to activate CreERT2.

### Statistical analysis

All data was obtained from at least three independent assays and shown as mean ± SEM. Prism 5.0 (Graphpad) was used to create graphs and to do statistics. Student’s t-test or ANOVA was used to detect the significance of differences, *p* < 0.05 was considered to be statistically significant. Only representative results are shown.

## Supplementary Information

**Additional file 1: Figure S1.** Sequence of the final targeting vector. Homology arms are in green. cKO region is in blue. Frt sites are in violet, LoxP sites are in red. Exon 4 and 5 are underlined. Sequence confirmed regions are highlighted in yellow. Sequence in lower-case letters is sequence deleted after Flp and Cre-mediated recombination.

**Additional file 2.**

## Data Availability

The datasets used and/or analysed during the current study is available from the corresponding author on reasonable request. The sequencing data is available in the NCBI SRA database: [https://www.ncbi.nlm.nih.gov/sra/PRJNA668444]. Accession ID: PRJNA668444.

## Abbreviations

PSMD11: Proteasome 26 s subunit, non-ATPase, 11
RPN-6: Regulatory particle non-ATPase 6
CP: Core Particle
RP: Regulatory Particle
BAC library: Bacterial Artificial Chromosome
hESCs: Human Embryonic Stem Cells
MEFs: Mouse Embryonic Fibroblasts
CMV: Cytomegalovirus
cKO: Conditional Knockout
AAV: Adeno-associated virus
Flp: Flippase
ACTB: Actin Beta
DAPI: 4,6-diamino-2-phenyl indole
RT-PCR: Reverse Transcription-quantitative Polymerase Chain Reaction
ANOVA: Analysis of Variance
SEM: Standard Error of the Mean
PIs: Proteasome Inhibitors
PARP: Poly ADP Ribose Polymerase
Cre: Cyclization recombination enzyme
Floxed: Flanked by LoxP
LoxP: Locus of X-overP1
frt: flippase recognition target
Neo: Neomycin
ZsGreen: Reef coral Zoanthus sp. green fluorescent protein
KO: Knockout
WT: Wild Type
SPF: Specific Pathogen Free
BSA: Bovine Serum Albumin
LSM: Laser-scanning microscopy
DTT: Dithiothreitol
ATP: Adenosine triphosphate
DNA: DeoxyriboNucleic Acid
PSMC 3: Proteasome 26S subunit, ATPase 3
PSMC 4: Proteasome 26S subunit, ATPase 4
PSMD 4: Proteasome 26S subunit, non-ATPase 4
siRNA: Small interfering RNA
RFU: Relative Fluorescence Units
SD: Standard Deviation
