# Supplementary material for: Generation and identification of a conditional knockout allele for the PSMD11 gene in mice
Source: BMC Dev Biol. 2021 Feb 1;21:4. doi: 10.1186/s12861-020-00233-1 (PMC7849139; doi:10.1186/s12861-020-00233-1)
Supplement: Supplementary file 1 — Additional file 1: Figure S1. Sequence of the final targeting vector. Homology arms are in green. cKO region is in blue. Frt sites are in violet, LoxP sites are in red. Exon 4 and 5 are underlined. Sequence confirmed regions are highlighted in yellow. Sequence in lower-case letters is sequence deleted after Flp and Cre-mediated recombination. [file 12861_2020_233_MOESM1_ESM.docx]

**Supplemental Information**

**Generation and identification of a conditional knockout allele for the PSMD11 gene in mice**

Linlin Zhao, Jinming Zhao, Yingying Zhang, Lele Wang, Longyan Zuo, Airu Niu, Wei Zhang, Xia Xue, Suhong Zhao, Chao Sun, Kailin Li, Jue Wang, Zhimin Bian, Xiaogang Zhao, Dieter Saur, Barbara Seidler, Chuanxin Wang, Tonggang Qi

**Institute of Medical Sciences, The Second Hospital, Cheeloo College of Medicine, Shandong University, Jinan, China, 250033**

Linlin Zhao, Jinming Zhao, Yingying Zhang, Chao Sun, Kailin Li, Jue Wang, Tonggang Qi

**Department of Clinical Laboratory, The Second Hospital, Cheeloo College of Medicine, Shandong University, Jinan, China, 250033**

Lele Wang, Chuanxin Wang,

**Department of Pathology, Liaocheng People’s hospital, Liaocheng, China, 252000.**

Longyan Zuo

**Department of Clinical Laboratory, Sanhe Yanjiao No.23 Hospital, Beijing, China, 065201.**

Airu Niu

**Department of Medical Imaging, The Second Hospital, Cheeloo College of Medicine, Shandong University, Jinan, China, 250033.**

Wei Zhang, Suhong Zhao

**Department of Pharmacy, The Second Hospital, Cheeloo College of Medicine, Shandong University, Jinan, China, 250033.**

Xia Xue

**Comprehensive Department, National Cancer Center/National Clinical Research Center for Cancer/Cancer Hospital, Chinese Academy of Medical Sciences and Peking Union Medical College, Beijing, China, 100021.**

Zhimin Bian

**Department of Thoracic Surgery/Key Laboratory of Thoracic Cancer in Universities of Shandong, The Second Hospital, Cheeloo College of Medicine, Shandong University, Jinan, China, 250033.**

Xiaogang Zhao

**The II. Medizinische Klinik und Poliklinik der Technischen Universität München, Ismaningerstr. 22, 81675 München, Germany.**

Dieter Saur, Barbara Seidler

| 1 | GTGGCACTTT TCGGGGAAAT GTGCGCGGAA CCCCTATTTG TTTATTTTTC TAAATACATT CAAATATGTA TCCGCTCATG AGACAATAAC CCTGATAAAT |
| --- | --- |
| 101 | GCTTCAATAA TATTGAAAAA GGAAGAGTAT GAGTATTCAA CATTTCCGTG TCGCCCTTAT TCCCTTTTTT GCGGCATTTT GCCTTCCTGT TTTTGCTCAC |
| 201 | CCAGAAACGC TGGTGAAAGT AAAAGATGCT GAAGATCAGT TGGGTGCACG AGTGGGTTAC ATCGAACTGG ATCTCAACAG CGGTAAGATC CTTGAGAGTT |
| 301 | TTCGCCCCGA AGAACGTTTT CCAATGATGA GCACTTTTAA AGTTCTGCTA TGTGGCGCGG TATTATCCCG TATTGACGCC GGGCAAGAGC AACTCGGTCG |
| 401 | CCGCATACAC TATTCTCAGA ATGACTTGGT TGAGTACTCA CCAGTCACAG AAAAGCATCT TACGGATGGC ATGACAGTAA GAGAATTATG CAGTGCTGCC |
| 501 | ATAACCATGA GTGATAACAC TGCGGCCAAC TTACTTCTGA CAACGATCGG AGGACCGAAG GAGCTAACCG CTTTTTTGCA CAACATGGGG GATCATGTAA |
| 601 | CTCGCCTTGA TCGTTGGGAA CCGGAGCTGA ATGAAGCCAT ACCAAACGAC GAGCGTGACA CCACGATGCC TGTAGCAATG GCAACAACGT TGCGCAAACT |
| 701 | ATTAACTGGC GAACTACTTA CTCTAGCTTC CCGGCAACAA TTAATAGACT GGATGGAGGC GGATAAAGTT GCAGGACCAC TTCTGCGCTC GGCCCTTCCG |
| 801 | GCTGGCTGGT TTATTGCTGA TAAATCTGGA GCCGGTGAGC GTGGGTCTCG CGGTATCATT GCAGCACTGG GGCCAGATGG TAAGCCCTCC CGTATCGTAG |
| 901 | TTATCTACAC GACGGGGAGT CAGGCAACTA TGGATGAACG AAATAGACAG ATCGCTGAGA TAGGTGCCTC ACTGATTAAG CATTGGTAAC TGTCAGACCA |
| 1001 | AGTTTACTCA TATATACTTT AGATTGATTT AAAACTTCAT TTTTAATTTA AAAGGATCTA GGTGAAGATC CTTTTTGATA ATCTCATGAC CAAAATCCCT |
| 1101 | TAACGTGAGT TTTCGTTCCA CTGAGCGTCA GACCCCGTAG AAAAGATCAA AGGATCTTCT TGAGATCCTT TTTTTCTGCG CGTAATCTGC TGCTTGCAAA |
| 1201 | CAAAAAAACC ACCGCTACCA GCGGTGGTTT GTTTGCCGGA TCAAGAGCTA CCAACTCTTT TTCCGAAGGT AACTGGCTTC AGCAGAGCGC AGATACCAAA |
| 1301 | TACTGTTCTT CTAGTGTAGC CGTAGTTAGG CCACCACTTC AAGAACTCTG TAGCACCGCC TACATACCTC GCTCTGCTAA TCCTGTTACC AGTGGCTGCT |
| 1401 | GCCAGTGGCG ATAAGTCGTG TCTTACCGGG TTGGACTCAA GACGATAGTT ACCGGATAAG GCGCAGCGGT CGGGCTGAAC GGGGGGTTCG TGCACACAGC |
| 1501 | CCAGCTTGGA GCGAACGACC TACACCGAAC TGAGATACCT ACAGCGTGAG CTATGAGAAA GCGCCACGCT TCCCGAAGGG AGAAAGGCGG ACAGGTATCC |
| 1601 | GGTAAGCGGC AGGGTCGGAA CAGGAGAGCG CACGAGGGAG CTTCCAGGGG GAAACGCCTG GTATCTTTAT AGTCCTGTCG GGTTTCGCCA CCTCTGACTT |
| 1701 | GAGCGTCGAT TTTTGTGATG CTCGTCAGGG GGGCGGAGCC TATGGAAAAA CGCCAGCAAC GCGGCCTTTT TACGGTTCCT GGCCTTTTGC TGGCCTTTTG |
| 1801 | CTCACATGTT CTTTCCTGCG TTATCCCCTG ATTCTGTGGA TAACCGTATT ACCGCCTTTG AGTGAGCTGA TACCGCTCGC CGCAGCCGAA CGACCGAGCG |
| 1901 | CAGCGAGTCA GTGAGCGAGG AAGCGGAAGA GCGCCCAATA CGCAAACCGC CTCTCCCCGC GCGTTGGCCG ATTCATTAAT GCAGCTGGCA CGACAGGTTT |
| 2001 | CCCGACTGGA AAGCGGGCAG TGAGCGCAAC GCAATTAATG TGAGTTAGCT CACTCATTAG GCACCCCAGG CTTTACACTT TATGCTTCCG GCTCGTATGT |
| 2101 | TGTGTGGAAT TGTGAGCGGA TAACAATTTC ACACAGGAAA CAGCTATGAC CATGATTACG CCAAGCTCGA AATTAACCCT CACTAAAGGG AACAAAAGCT |
| 2201 | GGAGCTCCAC CGCCCGGGCT GGTTCTTTCC GCCTCAGAAG CCATAGAGCC CACCGCATCC CCAGCATGCC TGCTATTGTC TTCCCAATCC TCCCCCTTGC |
| 2301 | TGTCCTGCCC CACCCCACCC CCCAGAATAG AATGACACCT ACTCAGACAA TGCGATGCAA TTTCCTCATT TTATTAGGAA AGGACAGTGG GAGTGGCACC |
| 2401 | TTCCAGGGTC AAGGAAGGCA CGGGGGAGGG GCAAACAACA GATGGCTGGC AACTAGAAGG CACAGTCGAG GCTGATCAGC GAGCTCTAGG ATCTGCATTC |
| 2501 | CACCACTGCT CCCATTCATC AGTTCCATAG GTTGGAATCT AAAATACACA AACAATTAGA ATCAGTAGTT TAACACATTA TACACTTAAA AATTTTATAT |
| 2601 | TTACCTTAGA GCTTTAAATC TCTGTAGGTA GTTTGTCCAA TTATGTCACA CCACAGAAGT AAGGTTCCTT CACAAAGAGA TCGCCTGACA CGATTTCCTG |
| 2701 | CACAGGCTTG AGCCATATAC TCATACATCG CATCTTGGCC ACGTTTTCCA CGGGTTTCAA AATTAATCTC AAGTTCTACG CTTAACGCTT TCGCCTGTTC |
| 2801 | CCAGTTATTA ATATATTCAA CGCTAGAACT CCCCTCAGCG AAGGGAAGGC TGAGCACTAC ACGCGAAGCA CCATCACCGA ACCTTTTGAT AAACTCTTCC |
| 2901 | GTTCCGACTT GCTCCATCAA CGGTTCAGTG AGACTTAAAC CTAACTCTTT CTTAATAGTT TCGGCATTAT CCACTTTTAG TGCGAGAACC TTCGTCAGTC |
| 3001 | CTGGATACGT CACTTTGACC ACGCCTCCAG CTTTTCCAGA GAGCGGGTTT TCATTATCTA CAGAGTATCC CGCAGCGTCG TATTTATTGT CGGTACTATA |
| 3101 | AAACCCTTTC CAATCATCGT CATAATTTCC TTGTGTACCA GATTTTGGCT TTTGTATACC TTTTTGAATG GAATCTACAT AACCAGGTTT AGTCCCGTGG |
| 3201 | TACGAAGAAA AGTTTTCCAT CACAAAAGAT TTAGAAGAAT CAACAACATC ATCAGGATCC ATGGCACGCG CTTCTACAAG GCGCTGGCCG AAGAGGTGCG |
| 3301 | GGAGTTTCAC GCCACCAAGA TCTGCGGCAC GCTGTTGACG CTGTTAAGCG GGTCGCTGCA GGGTCGCTCG GTGTTCGAGG CCACACGCGT CACCTTAATA |
| 3401 | TGCGAAGTGG ACCTGGGACC GCGCCGCCCC GACTGCATCT GCGTGTTCGA ATTCGCCAAT GACAAGACGC TGGGCGGGGT TTGCTCGACA TTGGGTGGAA |
| 3501 | ACATTCCAGG CCTGGGTGGA GAGGCTTTTT GCTTCCTCTT GCAAAACCAC ACTGCTCGAC ATTGGGTGGA AACATTCCAG GCCTGGGTGG AGAGGCTTTT |
| 3601 | TGCTTCCTCT TGAAAACCAC ACTGCTCGAT TTGTTAGCAG CCTCGAATCA ACCCGGGCGA TCCTAGGCGA TGAGATCTAG CTGTCGCGAC AATGGCTCAC |
| 3701 | CACTGTCAGT TTTAGGTAAC TCATGGCTTT CTGGAATACA ATATACTACA TACAGCACCT TGATCTCCTT CTTCTGAATA CTGTTTTGGG CTTGGTTTGC |
| 3801 | TGAGAACCCA TTCTCTCCTC ATGTTGTGTT TTCCTTCTCT CTCTTTTTTT AAAGATTTAT TTATTTATTA TATCTAAGTA CACTGTAGCT ATCTTCAGAC |
| 3901 | ACACCAGAAG AGGCTGTCAG ATCTCATTAC AGATGGTTGT GAGCCACCAT GTGGTTGCTG GGATTTGAAC TCAGGACCTT TGGAAGAGCA GTCACTGCTC |
| 4001 | TTAACCCCTG AGCCATCTCT CCAACCCCTG TGTTTTCCTT CTCTAATCCC ATCTCACTCA GGCTTACTCT CCTGGACATT TTTTTCTTGC TAATGTTGAT |
| 4101 | TTGATCTTAA CTTCTTCAGT GACCCACATT GTATTTTATA CATGTTATAG TTCTGATTTT TTTTACCTTA TATAGCAATT ATGACTTATC ATCTTTAATA |
| 4201 | TATTTTCATT GGGAATTACT CTATACCTTG AGGTAACTCA AAGCTTTCCT CATTCCCAGT ACACATCAAA AAAAAAATTT TTTTTTTGTT TTTCGAGACA |
| 4301 | GGGTTTCTCT GTATAGCCCT GGCTGTCCTG GAACTCACTT TGTAGACCAG GCTGGCCTCG AACTCAGAAA TCTGTCTGCC TCTGCCTCCC CAGTGCTGGG |
| 4401 | TCTAAAGGCG TGCACGCCAC CATGCCTGGC TTCATTTTTT TTTTAAACAT TTATGTGCAT AAGTGTCTTG TCTACATGTA TATATGTGCA CCATGTGTGT |
| 4501 | CCAGTACATG AAAAGGTCGG ATGAGAATGT TCGATCCTGT GAAGCTGGAG CTGCCATATA GGTGCTGGGA ACCAAACTCA GGTCTCCTGT GAGAGCTACC |
| 4601 | AGTTTGTTTA TTTATTTATT TGTTTGTTTG TTTGTATGTA TGTATGTGAG TACACTGTTC CTGTTCAGAT AGATGGTTGT GAGCCTTCAT GTGGCTGTTG |
| 4701 | GAAATTGAAT TAAGGAGCCC CACTCACTCA GTCCCTGCTT GCTCTGGCCC AAAGATTATA TTTATTATTA TATATAAGTT TTCAGATGTA CCAGAAGAGG |
| 4801 | GTGCCGGATC TCATTATAGG TGGTTGTGAG CCACCATGTG GTTGCTGGGA TTTGAACTCA GAACAAGAGC AGTCAGTGCC CTTACCCACT GAGCTATCTC |
| 4901 | GCCAGCCTGC CAGTTATCTT TAACAGCAGA GCTGTCTTTC TGGCCCCTAG AGTAGTTTGT CTTAAAAGAA CTACATAATT AGTACTTGAA GCAGAAGGAA |
| 5001 | TATATCAGGC CTTGGGTGTT CAGAAAATAC TTTCTTGATT TAAGATCTGA GGCTTTTTTT TTAACTTAAT GATTTTATAT GTATGGATGT TTTATGTACA |
| 5101 | TGTATGTCTG TGCACTAGGT AGTGTCCTCA GAGGTCAGAA GAGGTTGTTA AATCCTTGGG GACTGGAGTT AGACAGTTGT GAGCCCCTAT GAAGGTGCTG |
| 5201 | AATATTGAAC CTGGGTCCTC TAGAAGAGCA GCCAGTGCTC TTAACCAATT AGCCATCTCT CCAGCCCCTG GGTATGAGTT TATTTAATGG CTATATGAAC |
| 5301 | ATATCTCACT TTTCCTTGTC TAGGTGACTC AGAAGTTTTT GTTGTGGGGA AATTGATACA GGTTTTATTC TTGGTTCTTT AAATTTTTCT GGTAATGTGA |
| 5401 | AAACAGAGAA GAAAGCCATA GATAGTTCAC TGTTGTTGGG CTTGAGGAAG ACTAAGAACA TCATTAAGAG AGCTAGACTT GTTTGTCTTA AAGAGCTTTT |
| 5501 | TATCAATAAA ATGAGAGGGA GGGGTTGACA AAATGGCTTA GCTGGTAAAG ATGCCTGCTG CCAAACCTGA TGAGCTTATA TCCATCCTTG GAATCCACAT |
| 5601 | GGTAGAAGGA AACACTAAGT CACTCAGACA AAATAACTAC GTAGCAAGCT CCATGACCTT TAAGTTTTTT TCCTCTGTAA CCTGCACTGA TATGTAATAA |
| 5701 | TAATTTCATA ATATACTCAG TAACTGAAAA TAGATTCAAA TTCATTTATA AGGCACAGAA ACTTTCTTGT TCTAGGAGAG TGTCTGTAGT GACCTTAACT |
| 5801 | GGTCTTGGTT AAGGCAGAAA GAACTCTGGG GGCAGGGAAA GCTGATACGT GACTTTGTGA AAATTGTTTA ACATCTGGAT TATCAGGGAA ATAAAGGTGG |
| 5901 | GTGAGATGCT AGAGTAGATT TGACTATCTC TTGCTTAGGA CTATTGGAGT TTTAAAATTT TATGTGAATT GATGTTTTTG CCTGCATGCA TGTTTGTTTG |
| 6001 | TATGAGGGTA TTAGATCCCC TGGAACTGGA GTCTCATGTG GGTCCTGGGA ATTGAACTTG ATCCTTTGAA GAGCAGCCAG TGCTCTTAAC TGCTGAGCCC |
| 6101 | ATCTCTTCAA CCCCTTGGAG TATTTATTGA AGTCTTACAT AGTATAGAGG ACTATACCAA TTTCTCAGCA TTTATTTTGT ATTATTTGGT AAAGCACTCC |
| 6201 | AGGTAAAACT TATGGACATT TTCTTAGTCT AACAAGTCAT ATATGACTTA AGACTTGTAT TTTCCTTGTT AAAAATATTC AAAACAGCCA GGCGGTGGTG |
| 6301 | GCGCATGCAC GCCTTTAATT CCAGTACTCA GGAGCAGAAG CAGGTGGATC TCCCTGAGTT GGAGGCCAGC CTGGTCTACA GAGCTAGTTC TAGAACAGCC |
| 6401 | AAGGCTACAC AAAGAAACCC TGTCTCGAAA ACACAGAAAA ACAAAATTCA GAATATTTTC AGTTCATCCA TATCATGACT ATATCTTGAA TTTGGCTTTT |
| 6501 | CTTAACATAC TGCAAAACTT AACATACTGT AAGGTTGTTT TAGTTTAGTT TTTGTTCTGT GGAGGTCAGT TGTGTTTAGC ATTGGATTAG GTAGCCACCA |
| 6601 | AGGGGCTTCA GTGAGGAGTT AGAACTCATT AGCCTAGTCC TAGTGAATTG TCTCAGTGTT TTGTTTACTT CATGAGCTTG TCAGTCTCAT CATGCTAGAT |
| 6701 | TATTCTCTTC AGTTTTCCTC TCACACCATC TTTCAGAAGA ACTTTGAATT TACTTAAGAA CCCATTGTAT TAGCAGTTCT CCAGACAGTT CTGTTTCTCA |
| 6801 | TTTTACTGTC TTACTTTCTT ATTAACAATC TTTTTTTTTT TTAAAAGATT ATTTTATTTA TATGAGTACA CTGTCAGTGT CTTCAGATAT ACCAGAAGAT |
| 6901 | AGTATCAGAT GGTTGTGAGC CACCATGTGG TTCCTGGGAA TCGAACTCAG GACCTCTGGA AGAGCAGTCA GTGCTCTTAA CCACTGAGCC ATCTCTCCAG |
| 7001 | CCCCTATTTT CCCCTTTAAT CTAAGTTTAC TGTATTTGTT TTTCTGTGTG AATGATAACA GTATTTAAGA GAAAGAGCAA GCTAGATGTT TTGAGCCTTC |
| 7101 | CAGCTGTTAG CAATGAGGCT TTTCTTTTTT TATATTCTAG AAAATTTAAT ATTATCAAAT AATATCAGGA TTAATATTTC AAAGACAAGA AAATTTCTAT |

| 7201 | TATTTAAATT GTGAAGAAAA ATACTATATA TTTAGTTTAG AGAAAGGGTA ATTCTTTCTT TCAGAGATTA AATAAGCAGA ATTATTGGGT GCAATCTTAC |
| --- | --- |
| 7301 | ATCAATTTTA AATCTTTTAT GTGAATATGT GTATGTTTAT GCATTTATAT GCTCCCTGTG GGGTTGTCCA TGGAGTCCAA AGGCATCAGA TTCCCTGTGG |
| 7401 | CCTCAACTGG AGTCAAGTCA ACAGAGGAAG ACTTGTGCTG GCAACTGAAC GTGGGTCTTC TGGAAGACCA GAAGGTGCTG GTAGACTTTG CACCACCTCT |
| 7501 | CCAGCACTCC TGTTTTTTTT TTTTTAATTA GATATTTTCT TTATTTATAT TTCAAATGTT ATCCCCTTTC CTAGTTTTCT CTCCGAAAAT CCCCTATATC |
| 7601 | TCCCCCCCAC CCCCGGCTTT TTTTTTTTTT TTTTTTTTTT CTCGAGACAG GGTTTCTCTG TTTAGCCCTG GCTGTCCTGG AAATCACTTT GTAGACCAGG |
| 7701 | CTGGCCTTGA ACTCAGAAAT CTGCCTGCCT CTGCCTCCAG GCATGCGCCA CCATGTAAGG CTTTTTTAAT GTGTGGAGTT TGACCTCCTT TTACTAGCAT |
| 7801 | AGTCACAGTG TTTACATTAC TGGTCTCAAC TCTTCAGGGA GAGAGTTATG CTTTAAAGAT ACCCACGATG TCATGAAAAT AGATATTCCA GGACTATGTG |
| 7901 | TTGAGAAATT CTGAATATAC AGTAGGAAGG TAGAAGAGGA AGCTTTTTCC AAAGCACACT TTTTTTTTTT TTTTTTTGAG ACAGGGTTTC TATATATCCC |
| 8001 | TGGCTCATGG AACTCACTAT ATAGACCAGT CTGGCTGGCC TAGAACTTAT AGGGATTTGC CTGCTACTTT CTCCCAAATG TGGAACTAAA GGTGTGTACC |
| 8101 | ACCATGCTGG GCCAAAGAAT ATAAATTTAA AAAGCCAAGT TGCTTCTTGA TTACAGAGTA AGCATTACAT GTTGAAAATC TTGTGGGCCA GCATCCTATG |
| 8201 | GACTGTGTGA ACAACATGGG GTATCTGAAA GAAGGGAGTT AGAGCTTTCC TGCTTCTAAT TGTGGTTAGT TGGAAGAAGG TAAGGGCATC ATGTTACATG |
|  | E4 |
| 8301 | TATGTCTCTC TTGCAGGTCG AGCTATGTTT AGAGTGCATC GAATGGGCCA AATCAGAGAA AAGAACTTTC TTACGCCAAG CATTGGAGGT AGGTGCCTAC |
| 8401 | TTTGACATTG GCTGTATGAT CTGTAATGTT AGGTAAGATT TAAGTGTAAG TAGTACCACT CTGTTACCTA AGGGTTATTG ATAGATAGTG CTTCCAGTTA |
| 8501 | TGGGAGTGTC CCTGCTGGGA GTACTTTTCT AGTCTCAGTC TCAGTCTCTC TCTCTGTCTC TCTCTTTCTC TCTCTCTCCC TCCTCCCCTC CCTTCCTCCC |
| 8601 | TCTTCCCTCT CCTCAAGCTT GGCGCGCCTA GTGGATCCAC CTA**ataactt cgtatagcat acattatacg aagttat**att atgtacctga ctgat**gaagt** |
| 8701 | **tcctatactt tctagagaat aggaacttc**g aagggttccg caagctctag tcgagcccca gctggttctt tccgcctcag aagccataga gcccaccgca |
| 8801 | tccccagcat gcctgctatt gtcttcccaa tcctccccct tgctgtcctg ccccacccca ccccccagaa tagaatgaca cctactcaga caatgcgatg |
| 8901 | caatttcctc attttattag gaaaggacag tgggagtggc accttccagg gtcaaggaag gcacggggga ggggcaaaca acagatggct ggcaactaga |
| 9001 | aggcacagtc gaggctgatc agcgagctct agagaattga tcccctcaga agaactcgtc aagaaggcga tagaaggcga tgcgctgcga atcgggagcg |
| 9101 | gcgataccgt aaagcacgag gaagcggtca gcccattcgc cgccaagctc ttcagcaata tcacgggtag ccaacgctat gtcctgatag cggtccgcca |
| 9201 | cacccagccg gccacagtcg atgaatccag aaaagcggcc attttccacc atgatattcg gcaagcaggc atcgccatgg gtcacgacga gatcatcgcc |
| 9301 | gtcgggcatg cgcgccttga gcctggcgaa cagttcggct ggcgcgagcc cctgatgctc ttcgtccaga tcatcctgat cgacaagacc ggcttccatc |
| 9401 | cgagtacgtg ctcgctcgat gcgatgtttc gcttggtggt cgaatgggca ggtagccgga tcaagcgtat gcagccgccg cattgcatca gccatgatgg |
| 9501 | atactttctc ggcaggagca aggtgagatg acaggagatc ctgccccggc acttcgccca atagcagcca gtcccttccc gcttcagtga caacgtcgag |
| 9601 | cacagctgcg caaggaacgc ccgtcgtggc cagccacgat agccgcgctg cctcgtcctg cagttcattc agggcaccgg acaggtcggt cttgacaaaa |
| 9701 | agaaccgggc gcccctgcgc tgacagccgg aacacggcgg catcagagca gccgattgtc tgttgtgccc agtcatagcc gaatagcctc tccacccaag |
| 9801 | cggccggaga acctgcgtgc aatccatctt gttcaatggc cgatcccatg gtttagttcc tcaccttgtc gtattatact atgccgatat actatgccga |
| 9901 | tgattaattg tcaacaggct gcaggtcgaa aggcccggag atgaggaaga ggagaacagc gcggcagacg tgcgcttttg aagcgtgcag aatgccgggc |
| 10001 | ctccggagga ccttcgggcg cccgccccgc ccctgagccc gcccctgagc ccgcccccgg acccacccct tcccagcctc tgagcccaga aagcgaagga |
| 10101 | gcaaagctgc tattggccgc tgccccaaag gcctacccgc ttccattgct cagcggtgct gtccatctgc acgagactag tgagacgtgc tacttccatt |
| 10201 | tgtcacgtcc tgcacgacgc gagctgcggg gcggggggga acttcctgac taggggagga gtagaaggtg gcgcgaaggg gccaccaaag aacggagccg |
| 10301 | gttggcgcct accggtggat gtggaatgtg tgcgaggcca gaggccactt gtgtagcgcc aagtgcccag cggggctgct aaagcgcatg ctccagactg |
| 10401 | ccttgggaaa agcgcctccc ctacccggta gaatttcgac gacctgcagc caatccgcct cagaagccat agagcccacc gcatccccag catgcctgct |
| 10501 | attgtcttcc caatcctccc ccttgctgtc ctgccccacc ccacccccca gaatagaatg acacctactc agacaatgcg atgcaatttc ctcattttat |
| 10601 | taggaaagga cagtgggagt ggcaccttcc agggtcaagg aaggcacggg ggaggggcaa acaacagatg gctggcaact agaaggcaca gtcgaggctg |
| 10701 | atcagcgtca gatccgcctg ttgatgtagc tgctcaggta gtccagcacc tcctggctga tgatgccgtt ccaggcgggg tatctgatgc tgccctcggc |
| 10801 | gctgcccttc agctgctcga tgtgctgcca ctcctcgatg gggttggtct cgtccttcag ggcgatcatc tccttgctga tggggtcgta ggcgtagtac |
| 10901 | ctggacacca gggcgaagta gtggtcgggg atggcggtga tctggtgggt gtaggtggtc ctggccacgg cggaggccct cttgtcgctc cagttgccca |
| 11001 | ccacgtttgt cagctcggtc aggcccttca tgctcagaaa gctggtcatc aggtgcctgc cgatgtggct cttagggccg ttcttgatag cgaagatggg |
| 11101 | gtagggggcg ttcttcttca gggccttgtt gtagctgcgc accaggttgt ccttcagcag ctggtactcc tgcttgttgc tgctgctgtt gccggtcctg |
| 11201 | ttcactctct tcagcacggg ctcgctgttc ctcaggaact cgtccaggta caccaggggg tcgatcctgc ctctggcgct gaaaaagtag atgtgcctgg |
| 11301 | acacgcttgt cttggtctcg gtcaccaggc actgaatgat cacgcccagg tacttgttct gcaccagctt gaagctcttg gggtccacgt tcttgatgtc |
| 11401 | gctgaacctg ccgcagttga tgaatgtggc caggaacagg aactggtaca gggtcttggt cttggtgaac ctgctggtgt actcgaagct gttcaggatc |
| 11501 | ttctcggtga tctcccagat gctctcgccc tcggacagca gggccttcag catcttcttg ctgtggctgt tgcccttgtc ggcctcctcg ctgctctcga |
| 11601 | actgcagctg caggctggac acgatgtcgg tgatgtcgct ctggtgcttc tggccgttgt aagggatgat ggtgaactcc caggcgggga tcagcttctt |
| 11701 | caggctggcc tccaggatgg tggccttctg ggtcttgtac ttgaactgca ggctcttgtt cacgatgtcg aagctcaggc tgttgctgat gatggtgttg |
| 11801 | tagctcatga aggtggccct cttgatggcg gtgccgttgt gggtgatcat ccagcacagg taggtcagct cggcggcaca gctggcgatc ttctcgccgc |
| 11901 | tgggcctctc gaatctctcc acgaactgcc gcaccagcac cttggggggg gtcttgcaca ggatgtcgaa ctggctcatc accttcctct tcttcttagg |
| 12001 | agccatggcc gcaggaaagc agagccctga agctcccatc accggccaat aagagccaag cctgcagtgt gacctcatag agcaatgtgc cagccagcct |
| 12101 | gaccccaagg gccctcaggc ttgggcacac tgtctctagg accctgagag aaagacatac ccatttctgc ttagggccct gaggatgagc ccaggggtgg |
| 12201 | cttggcactg aagcaaagga cactggggct cagctggcag caaagtgacc aggatgctga ggctttgacc cagaagccag aggccagagg ccaggacttc |
| 12301 | tcttggtccc agtccaccct cactcagagc tttaccaatg ccctctggat agttgtcggg taacggtgga cgccactgat tctctggcca gcctaggact |
| 12401 | tcgccattcc gctgattctg ctcttccagc cactggctga ccggttggaa gtactccagc agtgccttgg catccagggc atctgagcct accaggtcct |
| 12501 | tcagtacctc ctgccagggc ctggagcagc cagcctgcaa cacctgcctg ccaagcagag tgaccactgt gggcacaggg gacacagggt ggggcccaca |
| 12601 | acagcaccat tgtccacttg tccctcacta gtaaaagaac tctagggttg cggggggtgg gggaggtctc tgtgaggctg gtaagggata tttgcctggc |
| 12701 | ccatggagct agcttggctg gacgtaaact cctcttcaga cct**gaagttc ctatactttc tagagaatag gaacttc**gga attcgatatc tctcgcaaca |
| 12801 | ttgtcttatt ctacagctca ggatgaaaac tttttttgac tcagtctact gaattctggg tttatagata tgagctatca tgcttgtact tggtggcttt |
| 12901 | cttttatatt attttgattt catcttgaag ttttaaggta cttttaattt tttgtataga aaataaacag cagtatggtg taggtaacag tcatgttgcc |
| 13001 | atagtaagaa atagtactgg ggctgagttc agttgttgtg ttgaacttga tgttattatc agatcttgta tagcacagta agtgttctgt gactgttaac  E5 |
| 13101 | cttttatttc tttttgtggc ttttcccctt gttccttgta atcaatgcct tttctctttc ctaggcaagg ctggtgtctt tgtattttga taccaagagg |
| 13201 | taccaggaag cattgcattt gggtaagtaa actggtagaa atcaaagtaa gacctctaaa ctagccaaat gagtggattc caaacctggt tattcttact |
| 13301 | gattgttaat aattatattt tacttgtgtc taagaagtat ttcaagtagt ttacaattca taaatttaca aatttataaa atttgtcacg gtaaagtcct |
| 13401 | gctttagatt cgtgaaaaac tggggtggag gtacagctca gtacacctgg gagtagcatg ggaatacatt tctgggaagc cagctgggct taatctcagg |
| 13501 | tttacagaat agacatcagt tttacgcttt aatttaaagt aggtcatttc attgaaatag tagctgattt tttttgtttt taagtttagt ttatacatac |
| 13601 | tatcatggta aatttcagat ttaatttttt taattacaaa tgttttatag actactataa agctactata tattttttgt aatttttaat ttttatttta |
| 13701 | tgtgcattgg tgttttaact gcatgtatgt ctgtgagggt gttggatact catcgatgct cgtacgcagt a**ATAACTTCG TATAGCATAC ATTATACGAA** |
| 13801 | **GTTAT**ACGTG TCGACCATAT GAGGAACGTG AGTTACAGAC GGTTGTGAGC TGCCATGTGG ATGCTAGGAA TTGAACCTGG GTCCTCTGGA AGAGTAGCCG |
| 13901 | ACACTCTTTA ATTGCTGAGC CATCTCTCTA TCCCCCTGTA TATTATTTTT TAATATTCAT TTTTATGTGT ATATGTCTAT GTGAGTATTT TGACATCTGT |
| 14001 | GCATACTCAT GGAAATCAGA AATGTTGGAT CCCTGTAGCT GAAGTTATAA ATAGTTGTGA GCCGCCTGAT GTAGGTGTGG GATCTGAGCT CACAGGTCAT |
| 14101 | CAGGAAGAGC ATTAACTTAA CATAATCTTA ACTGCTGAGC TATCTTTCCA TCCCCCCTCC CAATATTACT ATTTGTAATA TATTTAAAAT TTTCCTTTTT |
| 14201 | TGTTACGTTT TTATATTGGT GGGGTGAGGG AGAATGCATG TATGCACAGT GTGTATGTGG CGATCAGAGA ATAGTTCTAG GGAGTTGATT CTATTCTTCT |
| 14301 | AACTTAAGAA TTCTGGATTG AACTCAGGCT TGGTGACAGG CCATCTTGCT GGCCTTTAAA GTCTCTTAAA ATTTGAAAAT ATTGCTGGTC ATAGTGGCTC |
| 14401 | AAGCCTTGGT CAAAAGTCCA AAATGGGCAT GGCTTAGTTG GCAAAACGCT TGCTGTGCAC ACACAAGGAT CTGAGTTTGG TTCCTAGAAC TCAAGTACAA |
| 14501 | GGCCTCCAAT CCCATTACTG GGAGACAGAG ACAGAAGAAT CACTGAGGCT GACTGGTCAG TCAGCCGTGT TCTAACCTGT GAGGCAGCCC CAAGTCTCAT |
| 14601 | TGAAAGCTCC TGTCTCAAGA ACACAAGGTG TACAGCTCCA GAGGAGCACC TGAAATTAGT GGTGGCAAGT TGTATAACTT AGTGAAGATT CTGAAACAAT |
| 14701 | CTACTGCATT ATATCATTTT GATAAGGTGA GTTCTGATGA CTGGGGAGGT AGCTCAGTTT GTTAAATGCC ATGAGGACTT GAAGACCTGA GTTTGATCCT |
| 14801 | CAGAACCCAG GTAAAAAGCC AGGTGTGGTA ACAGACTTCT AATTCCAGTA CTGCAGCGGG AGAGAGAGAG AAAGGAAGAT CCCCAGGGAG GAAAAGGCGG |
| 14901 | AGAGAGCAGA GAGAACTGAC CCATCACTAC AAGTTGACCT GGTTTACACA CACACACACA CACACACACA CACACACACA CACACACACA CACACTCACA |
| 15001 | CACTCACACA CACTCACACA CACTCACACT CACTTACTCT GGGCCGGGGG GGGGGGGGAG GTGTTAGGAA ACTGAGTACT TATATTTTTG GAAGCTGGGT |
| 15101 | GTTTTTTATT GTTTTAATGT TTTTTTTTTT ATTTTAAGAT TTATTTATTT ATTATATATA CATTGACTAG CTGTCTTCAG ACACACCAGA AGAGGGCATC |
| 15201 | ATTAGGGATG GTTGTGAGCC ACCATGCAGT TGTTGGGATT TGAACTCAGG ACCTCTAGAA GAGTAGTCAG TGCTCTTAAC TGCTGATCCA TCTCTCCAGC |
| 15301 | CCCCATTGTA ATGTTTTTAT TAGCATATAT GATTACCTAT GATGAGTTTC ACTATTACAT TTTCATAGAG ATATGTAATG TAGGTCAGTC GTACTCAACT |
| 15401 | CCTCTTGTTA CCCTTTTTTA TATTTCCCCC TGTGCTAGTC GCTTTCCTTG CTCCAATTAT TAGCTCTTAT GTTTTCCTTT GTGATCCAGT GGATTAAGGC |
| 15501 | TGTTGTTAAC ACCATGGATA AGAGTTTGTA GGACTATGAA CACCTCACAG TAGGTACATC ATTGAAGAAG ACATTCTTTT TCCCTGATCA GTCATTGGTG |
| 15601 | CCAGTTAAAT CCTCCTCATA TGATCTTTAA ATACCCTAGA AATCTCCCAT TGCTTGCTTC TGGATTTATT TTATGTGTGT AAGTGTTTAA CTTGCATGTG |
| 15701 | TTGTGTGTGT ACACTGTGTG CATGCCTATG GGTGGTATAA GTCACCATGT AGATACTGGG AACTGAACCT GGGTTCTCTG TAAGAACAGA GTGTTCTTAA |
| 15801 | CCACTGAGCC TTCTTTCCAG TTACCTTTTT TTGTTTTGTT TTGTTTTGTT TGGTTTTCAA GACAGGGTTC CTCTGTGTAG CCCTGGCTGT CCTGGAACTC |
| 15901 | ACTCTGTAGA CCAGGTTGGC CTCTAACTCA GAAATCTGCC TGCCTCTGCC TCCCATGTGC TGGGATTAAA GGTGTGTGCC ACCACTGCCC AGCTATACTT |

| 16001 | TTTTTGAGAC | AGGTTTTTAT | AGGTTCAGGC | TTCTGCTTTA | GCCTCCCTCC | TGTTAGAATT | ACAGGCACAT | GGTATCATGC | TATCTTTACT | TGCTCTTTAT |
| --- | --- | --- | --- | --- | --- | --- | --- | --- | --- | --- |
| 16101 | AAATAATAAT | ATCGCATTTC | CCCTATGATT | TATTAAGTTG | AAAATTGCAG | GGAACCTTGG | ATTTGGCTTG | GCCTAGCTGC | CTTGAGAGGC | ATGTGTCATA |
| 16201 | GGTATTTCAA | AGTATTCACA | CTGACTTTTT | AGCACCCTGA | GAAAAAACAA | ACATAGCCTT | GGCTCCAGTG | ACTTACTGGA | TCTGGGTCAT | TCAATTGGCC |
| 16301 | ACACAAAATG | TCTCTCCAAA | GGACTGTGAC | TTTGAATATT | TTTGGAGGGG | CTCAAAAACG | TTCTGCAGAA | GTAAGACCAT | TCTTCCCCAA | ATTAAAAAAA |
| 16401 | TTGTACTGTA | TATCTAGAAG | ACAAAATATT | GTGTGGAGTA | GCCAGCCATA | CTGCCCTGCT | CCCCCTCCTT | TGTTTTTAAA | TAACATTTAA | GTTGTTCATT |
| 16501 | ATTTCTTTTT | ACAAAAGATT | TACCCTGAGG | ATTTTAAAGA | ATGTCTATGA | TATGGGTTAT | ATCAAACTTT | TGGCAAAATT | TCTGAATAGG | CTTGTATGTA |
| 16601 | TGTAGGAGCA | TTGTTTTGTT | TGTTTGTTTG | GTTTTGGTTT | TTCAAGACAG | GGTTTCTCTG | TGTAGCTCTC | CTGGCGGCCG | CGTACCCAAT | TCGCCCTATA |
| 16701 | GTGAGTCGTA | TTACAATTCA | CTGGCCGTCG | TTTTACAACG | TCGTGACTGG | GAAAACCCTG | GCGTTACCCA | ACTTAATCGC | CTTGCAGCAC | ATCCCCCTTT |
| 16801 | CGCCAGCTGG | CGTAATAGCG | AAGAGGCCCG | CACCGATCGC | CCTTCCCAAC | AGTTGCGCAG | CCTGAATGGC | GAATGGGACG | CGCCCTGTAG | CGGCGCATTA |
| 16901 | AGCGCGGCGG | GTGTGGTGGT | TACGCGCAGC | GTGACCGCTA | CACTTGCCAG | CGCCCTAGCG | CCCGCTCCTT | TCGCTTTCTT | CCCTTCCTTT | CTCGCCACGT |
| 17001 | TCGCCGGCTT | TCCCCGTCAA | GCTCTAAATC | GGGGGCTCCC | TTTAGGGTTC | CGATTTAGTG | CTTTACGGCA | CCTCGACCCC | AAAAAACTTG | ATTAGGGTGA |
| 17101 | TGGTTCACGT | AGTGGGCCAT | CGCCCTGATA | GACGGTTTTT | CGCCCTTTGA | CGTTGGAGTC | CACGTTCTTT | AATAGTGGAC | TCTTGTTCCA | AACTGGAACA |
| 17201 | ACACTCAACC | CTATCTCGGT | CTATTCTTTT | GATTTATAAG | GGATTTTGCC | GATTTCGGCC | TATTGGTTAA | AAAATGAGCT | GATTTAACAA | AAATTTAACG |
| 17301 | CGAATTTTAA | CAAAATATTA | ACGCTTACAA | TTTA |  |  |  |  |  |  |

**Figure S1.** Sequence of the Final Targeting Vector. Homology arms are in green. cKO region is in blue. Frt sites are in violet, LoxP sites are in red. Exon 4 and 5 are underlined. Sequence confirmed regions is highlighted in yellow. Sequence in lower-case letters is sequence deleted after Flp and Cre-mediated recombination.
