# Supplementary material for: Generation and identification of a conditional knockout allele for the PSMD11 gene in mice
Source: BMC Dev Biol. 2021 Feb 1;21:4. doi: 10.1186/s12861-020-00233-1 (PMC7849139; doi:10.1186/s12861-020-00233-1)
Supplement: Supplementary file 2 — Additional file 2. [file 12861_2020_233_MOESM2_ESM.docx]

**Supplemental Information**

**Generation and identification of a conditional knockout allele for the PSMD11 gene in mice**

Linlin Zhao, Jinming Zhao, Yingying Zhang, Lele Wang, Longyan Zuo, Airu Niu, Wei Zhang, Xia Xue, Suhong Zhao, Chao Sun, Kailin Li, Jue Wang, Zhimin Bian, Xiaogang Zhao, Dieter Saur, Barbara Seidler, Chuanxin Wang, Tonggang Qi

**Institute of Medical Sciences, The Second Hospital, Cheeloo College of Medicine, Shandong University, Jinan, China, 250033**

Linlin Zhao, Jinming Zhao, Yingying Zhang, Chao Sun, Kailin Li, Jue Wang, Tonggang Qi

**Department of Clinical Laboratory, The Second Hospital, Cheeloo College of Medicine, Shandong University, Jinan, China, 250033**

Lele Wang, Chuanxin Wang,

**Department of Pathology, Liaocheng People’s hospital, Liaocheng, China, 252000.**

Longyan Zuo

**Department of Clinical Laboratory, Sanhe Yanjiao No.23 Hospital, Beijing, China, 065201.**

Airu Niu

**Department of Medical Imaging, The Second Hospital, Cheeloo College of Medicine, Shandong University, Jinan, China, 250033.**

Wei Zhang, Suhong Zhao

**Department of Pharmacy, The Second Hospital, Cheeloo College of Medicine, Shandong University, Jinan, China, 250033.**

Xia Xue

**Comprehensive Department, National Cancer Center/National Clinical Research Center for Cancer/Cancer Hospital, Chinese Academy of Medical Sciences and Peking Union Medical College, Beijing, China, 100021.**

Zhimin Bian

**Department of Thoracic Surgery/Key Laboratory of Thoracic Cancer in Universities of Shandong, The Second Hospital, Cheeloo College of Medicine, Shandong University, Jinan, China, 250033.**

Xiaogang Zhao

**The II. Medizinische Klinik und Poliklinik der Technischen Universität München, Ismaningerstr. 22, 81675 München, Germany.**

Dieter Saur, Barbara Seidler

**Full-lengh of blots and gels of figure 1.**

**d**

M 1 2 3 4 5 M

**
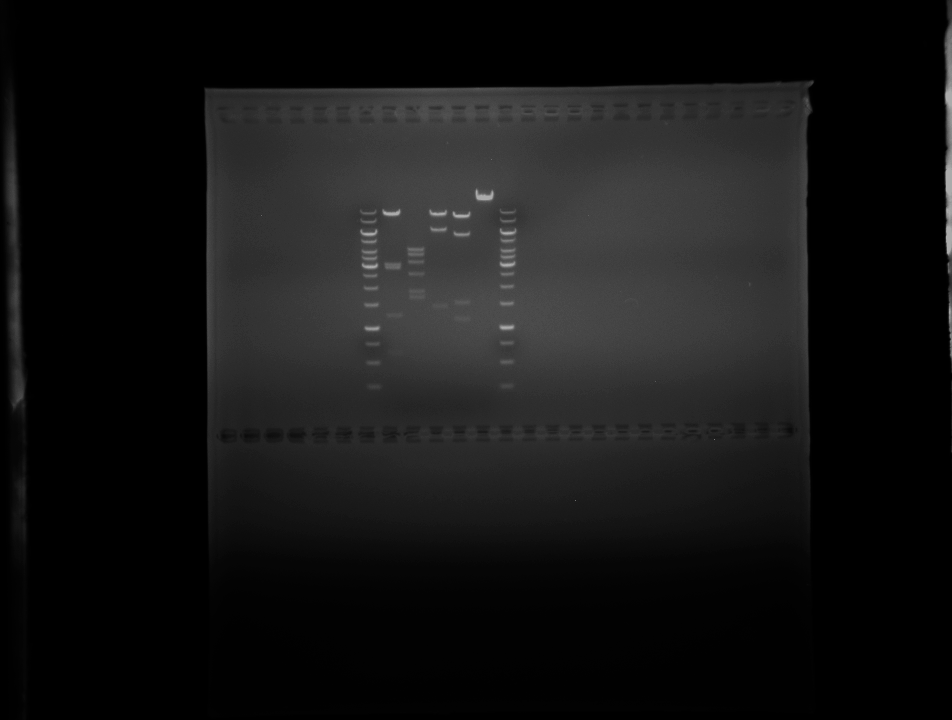

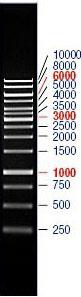
**

1. ApaLI : 9.3/3.1/2.9/1.2/0.6/0.1

2. DrdI : 4.2/3.8/3.3/2.6/1.9/1.6

3. EcoRI : 9.3/6.5/1.5/0.1

4. FspI : 8.9/5.7/1.5/1.2

5. NotI : 17.3

**Figure 1.** Construction and identification of a Conditional Targeting Vector For PSMD11. **a** Schematic figures of the wild-type allele of PSMD11, the targeting vector, PSMD11*^neo-flx^*（recombinant allele）, PSMD11*^flx^*(conditional floxed allele after Flp recombination ) and PSMD11^∆^(constitutive KO allele after Cre recombination) alleles. Blue boxes, Brown triangles and purple triangles represent exons with the exon number indicated, *loxP* sites and *frt* sites separately. The 5´ long homology arm (LA) including exon 4 of the targeting vector is∼4.93kb, the 3´ short homology arm (SA) is∼2.85kb. Small Red line denote the location of Neo probe for Southern blot. Arrows show the location of the primers for sequencing and genotyping. The F1 PSMD11 *^flx-neo/+^* mice was crossed with the FLPe deleter mice to remove the *Neo* cassette in the germline. PSMD11*^flx/flx^* mice were mated with CMV-Cre mice to generate PSMD11^∆/+^ mice. **b** Comparison of mRNA transcripts from PSMD11 exon 5 floxed allele or deleted allele to show the introduction of a stop codon (*) after removal of the E5 in the chimeric transcript of E4 and E6. The predicted amino acids are shown below the mRNA labeled in colors. **c** and **d** Restriction endonuclease pattern and analysis of the targeting vector which was digested with restriction endonuclease ApaLI，DrdI，EcoRI，FspI5 or NotI respectively, the DNA fragments was separated by 0.5% gel electrophoresis.

**Full-lengh of blots and gels of figure 2.**

a

Region 1 (WT: N.A.; MT:6.0 Kbp)

1G1 1D5 1B8 1D10 1B112H4 2H7 M WT

**
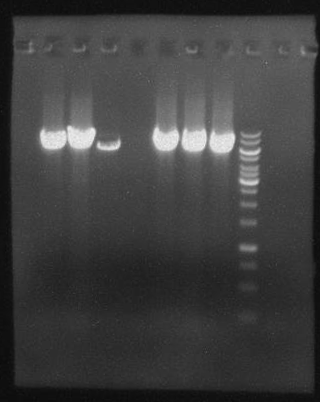
**

Region 2 (WT: 240 bp; MT: 310 bp)

1G1 1D5 1B8 1D10 1B11 M water WT

2H4 2H7M water WT

**
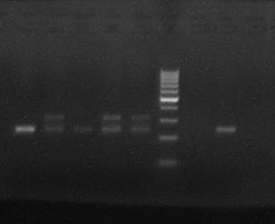

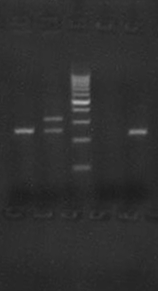
**

b

WT 1B11 1D5 2H7

WT 1B11 1D5 2H7


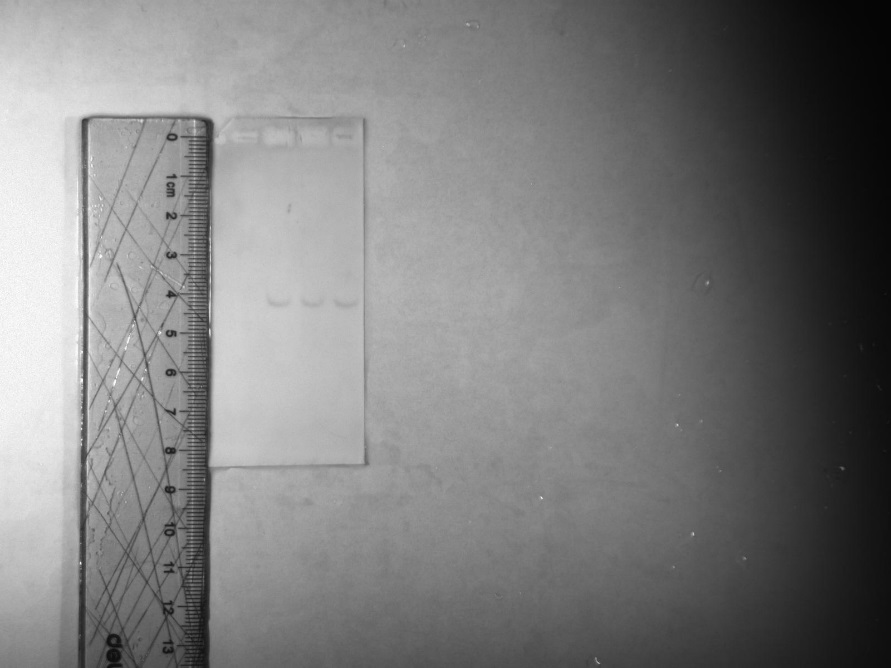

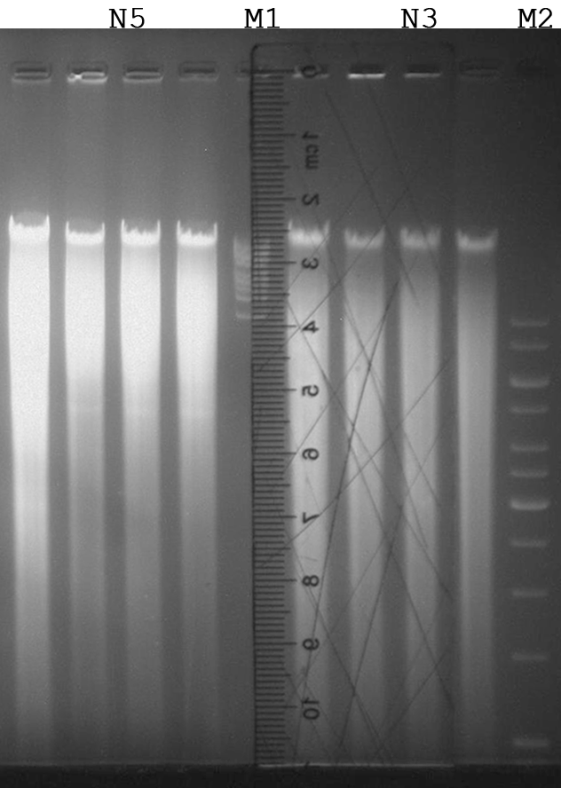

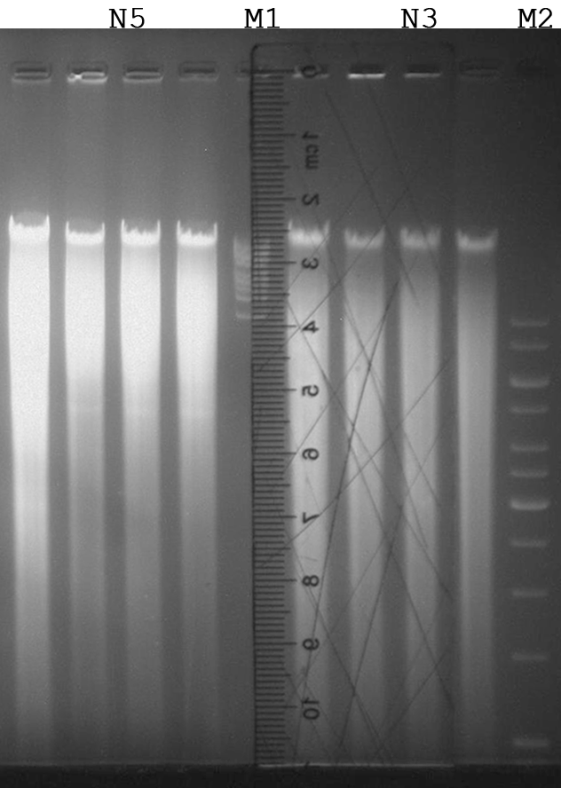

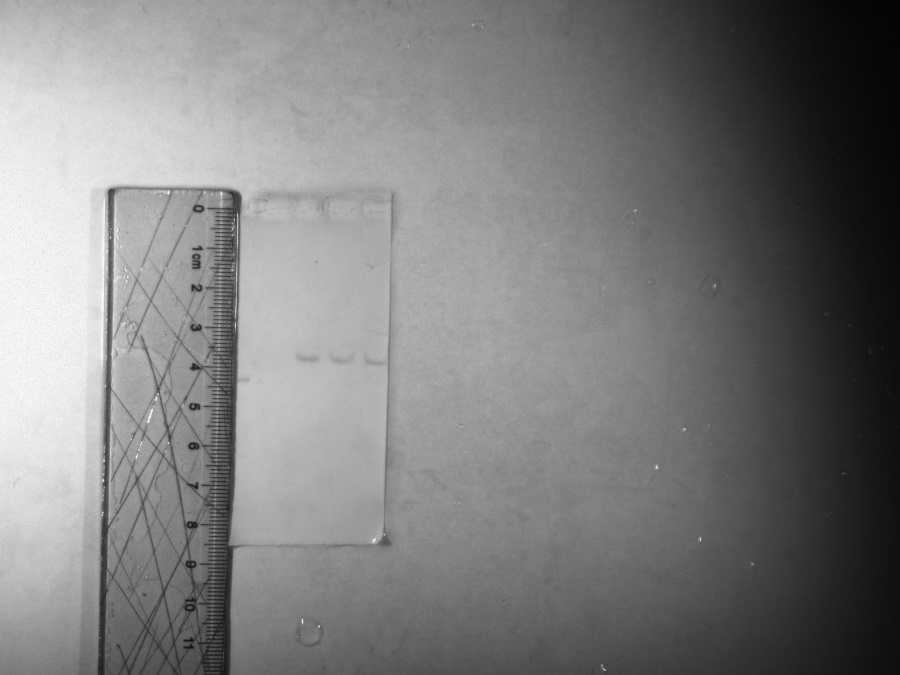


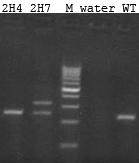

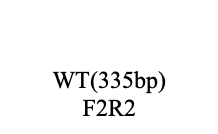

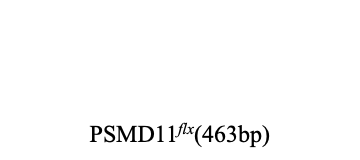


**5’-HA (10.1 Kbp; Nde I)**

**3’-HA (8.3 Kbp; Hind III)**

**bp**

**bp**

**bp**

3’- Homology Arm

(8.3 Kbp; Hind III)

5’-Homology Arm

(10.1 Kbp; Nde I)

*flx/flx* +/+ +/+ *flx*/+

M

c


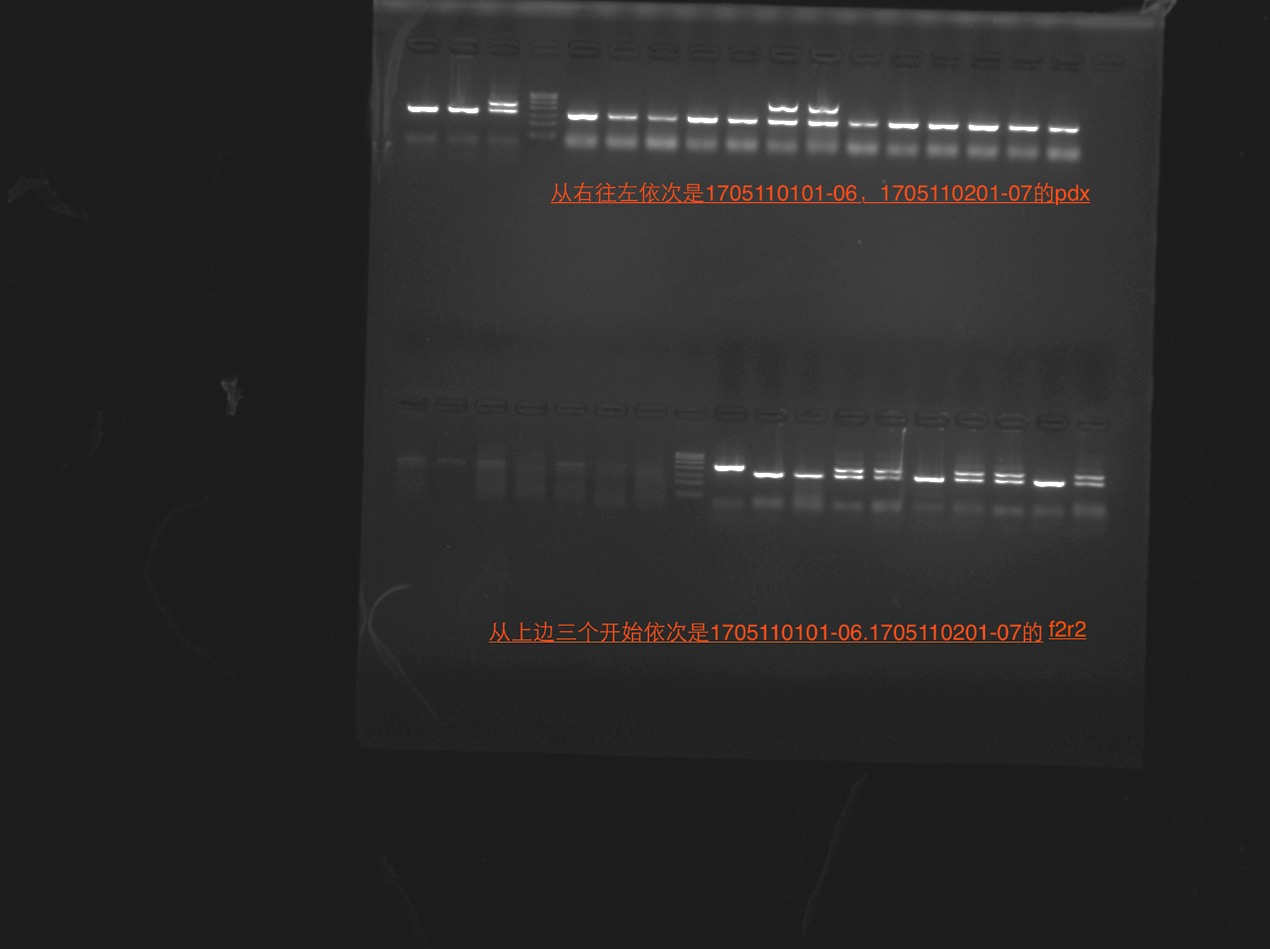


**PSMD11*^flx^*(463bp)**

**WT(335bp)**

**F2R2**

**Figure 2.** Generation of mice with floxed allele of PSMD11. **a** PCR amplification of region 1 and 2 with primer A1 and A2, or B1 and B2 to identify appropriately targeted embryonic stem cells. Size of the resulting PCR products was 6kb (MT) for primer A1 and A2, and 310bp (MT,) and 240bp (WT) for primer B1 and B2 respectively. **b** reconfirmation of targeted embryonic stem cell clones by southern blotting. Nde I and Hind III digested DNA from three individual clones that had passed all controls was electrophoretically separated on a 0.5% agarose gel, after transfer to a nylon membrane, a probe targeted against the Neo cassette was hybridized with the digested DNA, which showed two bands at 10.1 kb, and 8.3 kb respectively. **c** Genotyping of mice with genomic DNA from tail biopsies. Primers F2 and R2 were used to distinguish the floxed allele (PSMD11*^flx^*, 463bp) from the wild-type allele(WT, 335bp).

**Full-lengh of blots and gels of figure 3.**


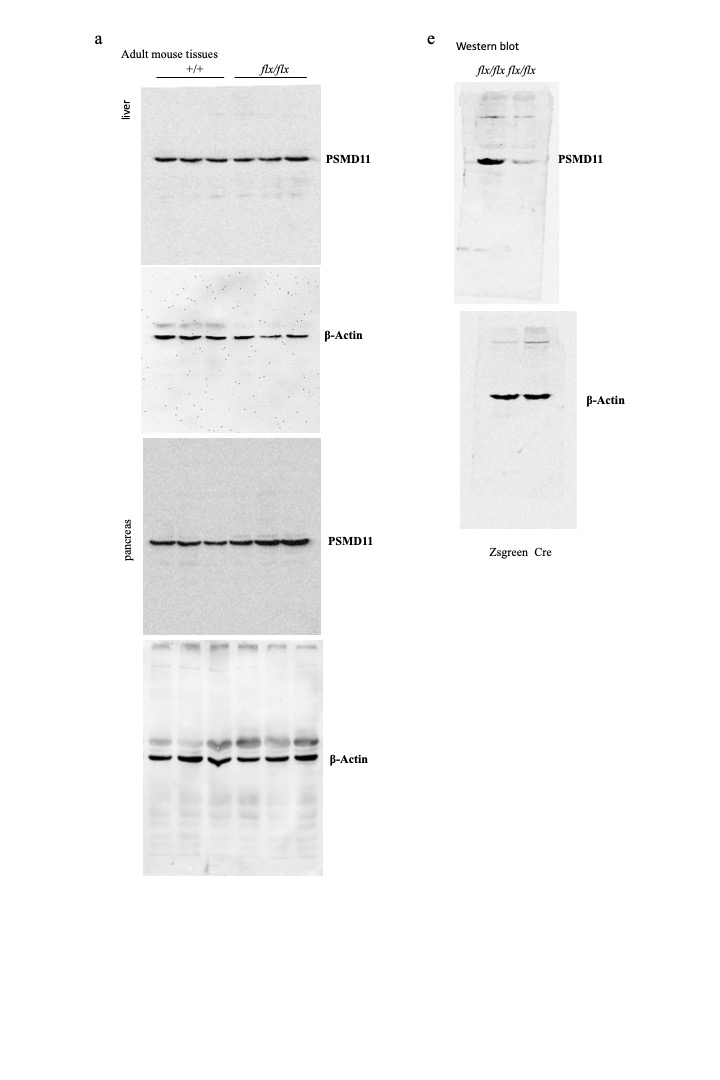


c

*flx/flx* MEFs, genotyping

Zsgreen

Cre

NC

M

*flx/flx flx/flx. flx/+*


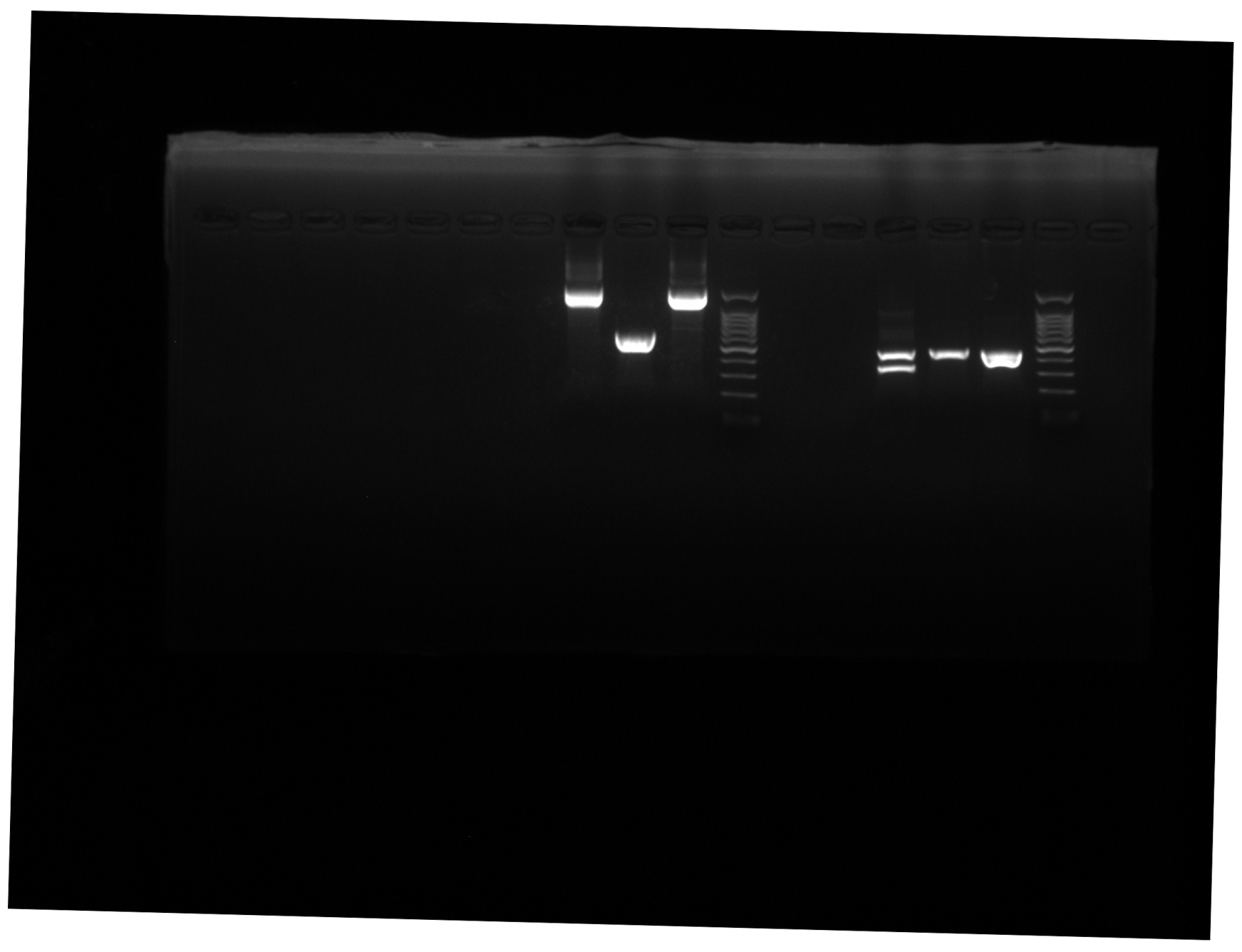


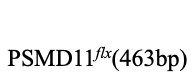


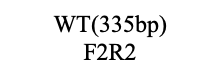


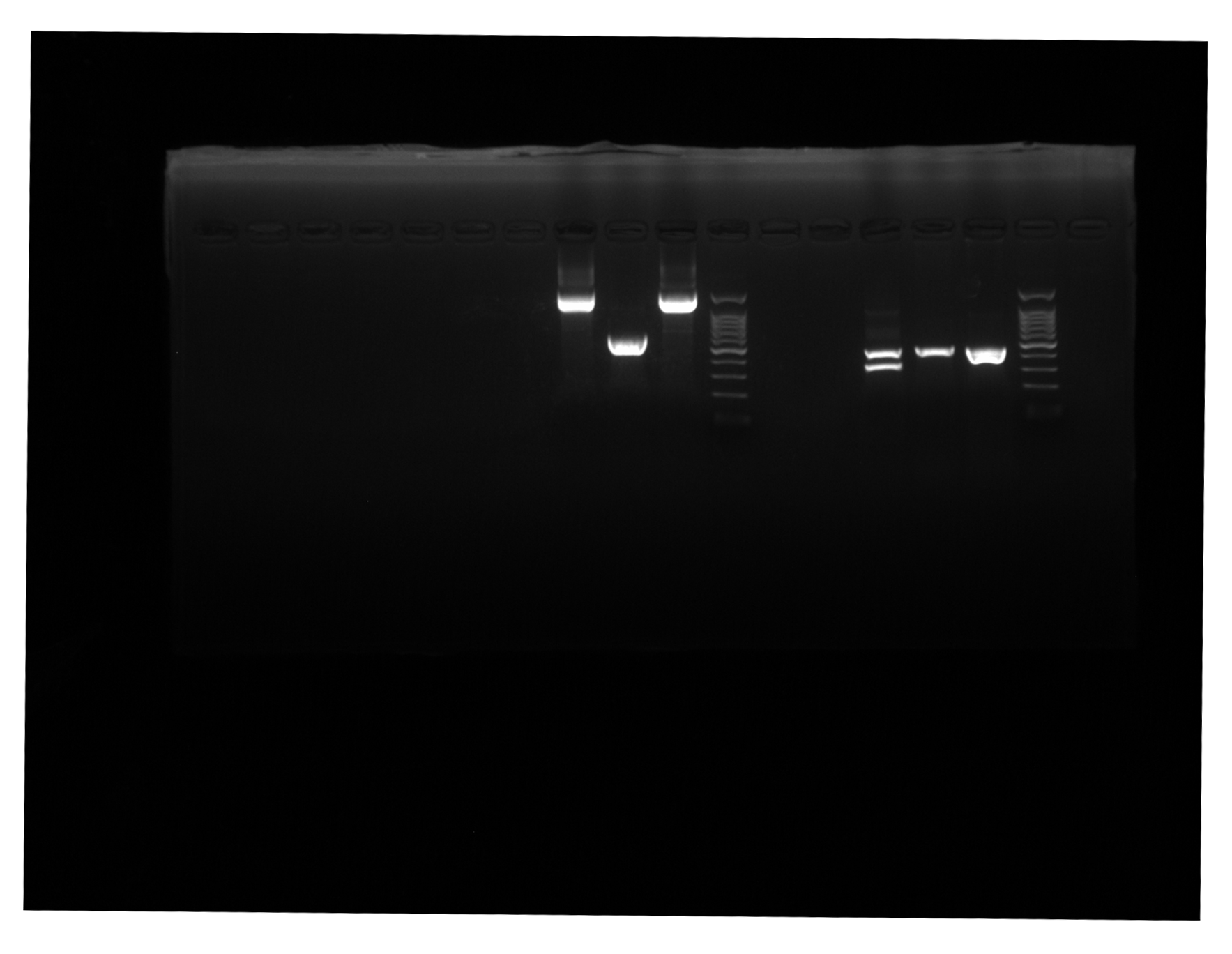


PSMD11*^flx/flx^* (1722bp)


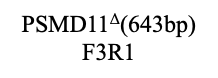


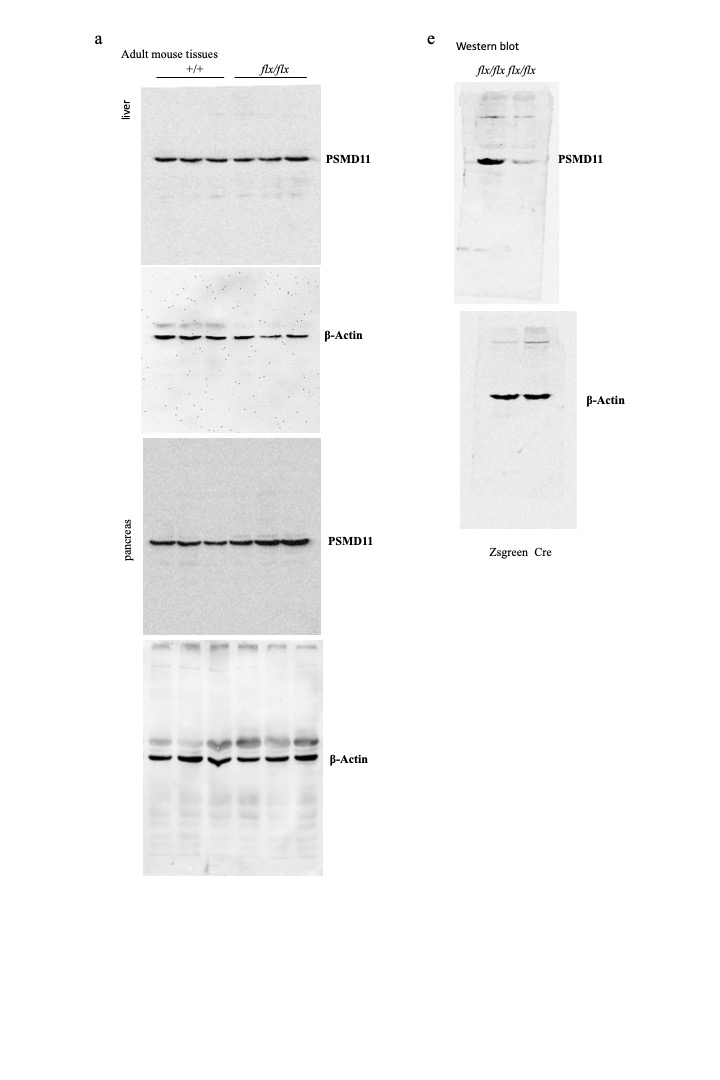


**Figure 3.** Floxed PSMD11 allele could be deleted efficiently in vitro with Cre-mediated gene recombination**. a** No difference of PSMD11 expression could be found in liver and pancreas between adult WT and PSMD11*^flx/flx^* mice by western blot. n=3 each group. **b** qRT-PCR to detect PSMD11 mRNA expression in RNA from adult WT and PSMD11 *^flx/flx^* mouse liver and pancreas tissues, n=3 each group. **c** PCR analysis of DNA from AAV (expressing ZsGreen or Cre) infected PSMD11 *^flx/flx^* MEFs and a nonrecombined PSMD11 *^flx/+^* mouse without Cre expression (NC), primer F3 and R1 will produce 643bp bands in PSMD11 exon 5 deleted alleles, primers F2 and R2 will produce 463bp and 335bp bands in PSMD11 exon 5 floxed or WT alleles respectively. **d** Quantification of PSMD11 mRNA by real-time RT-PCR on total RNA extracted from AAV (expressing ZsGreen or Cre) infected PSMD11 *^flx/flx^* MEFs. **e** Western blot to show nearly complete depletion of PSMD11 protein in Cre-infected PSMD11 *^flx/flx^* MEFs. **f** Quantification of PSMD11 protein expression in **e** which was normalized to β-Actin and expressed as percent of the controls. The mean ± SEM of two independent experiments was shown. ***P* < 0.01, **P* < 0.05.

**Full-lengh of blots and gels of figure 4.**

**
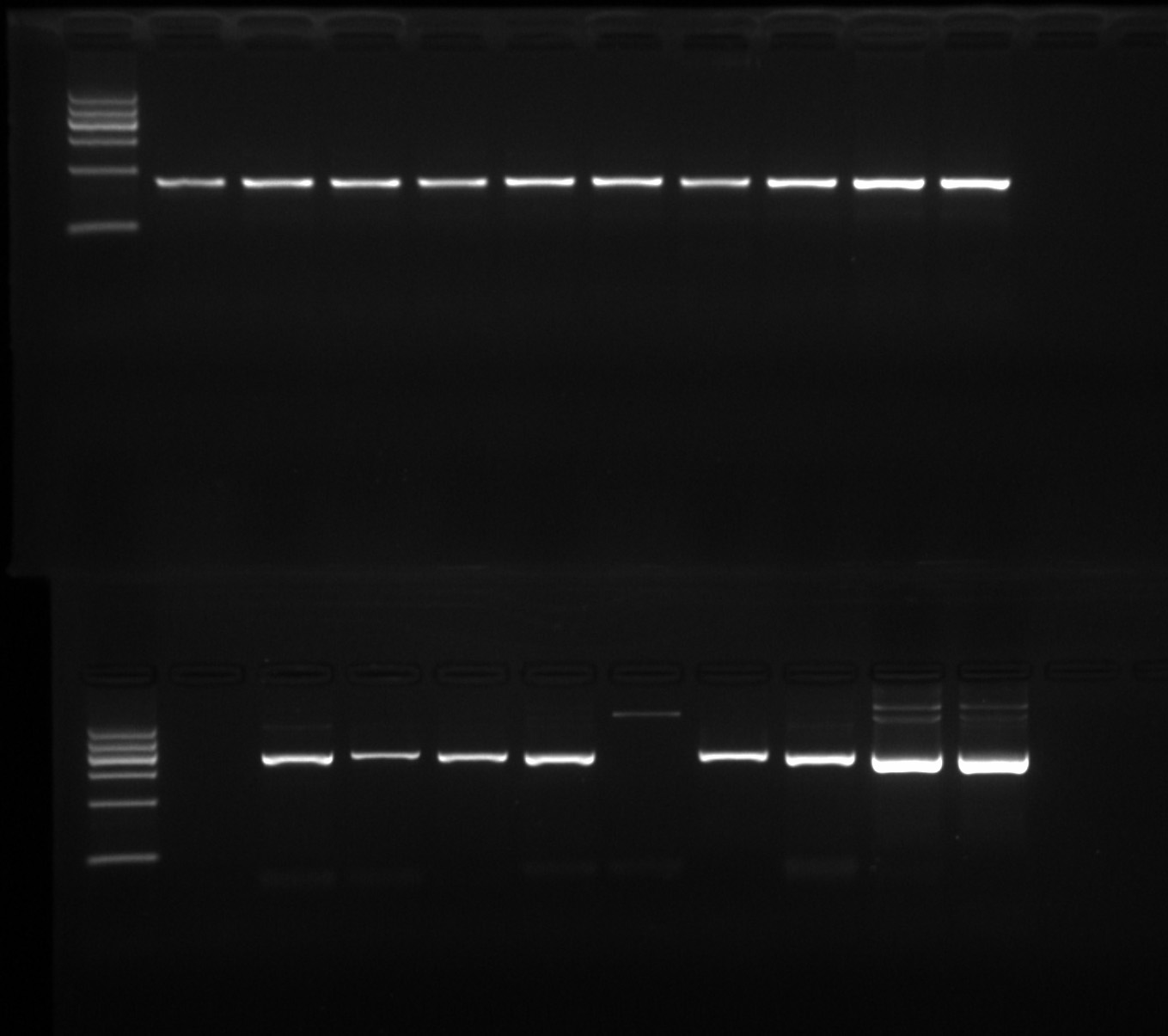
**

+/+ +/- +/- +/- +/- +/+ +/- +/- +/- +/-

F1/R1

F3/R1

b

PSMD11^∆^ (643bp)

WT(240bp)

**Figure 4.** Constitutive ablation of PSMD11 leads to early embryonic lethality (PSMD11^-/-^) and growth retardation (PSMD11^+/-^) in mice. **a** Methods to generate PSMD11 constitutive KO mice. **b** Genotyping of E7.5-8.5 embryos from intercrossing between PSMD11^+/-^ heterozygous mice. The 643bp band indicate the exon 5 deleted allele of PSMD11 (PSMD11^∆^). **c** Representative Smaller body size of PSMD11^+/-^ mice compared with WT littermates. **d** Growth curve of PSMD11^+/-^ mice (n=9) and age- and sex-matched male and female WT littermates (n=9), **P < 0.01, *P < 0.05. **e** Representative image of E7.5-8.5 embryos of mice under stereomicroscope. **f** Representative HE images of E7.5-8.5 WT and PSMD11^+/-^ embryos of mice. **g** and **h** Representative images of Arcturus Histogene^TM^ staining and Laser Capture Microdissected tissues of E7.5-8.5 embryos of mice.

**Full-lengh of blots and gels of figure 5.**

M

**b**

- + + - - + - - + +

**
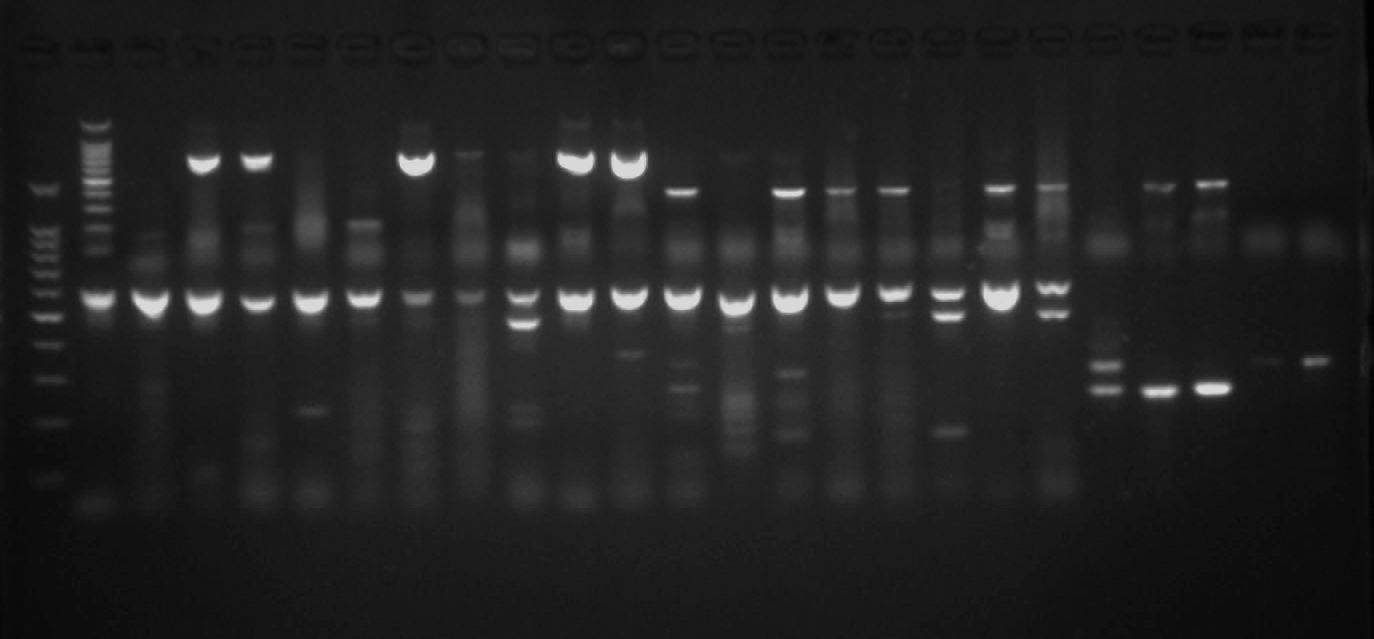
**

*Flp* 725bp

- + + + - - + - + +

**
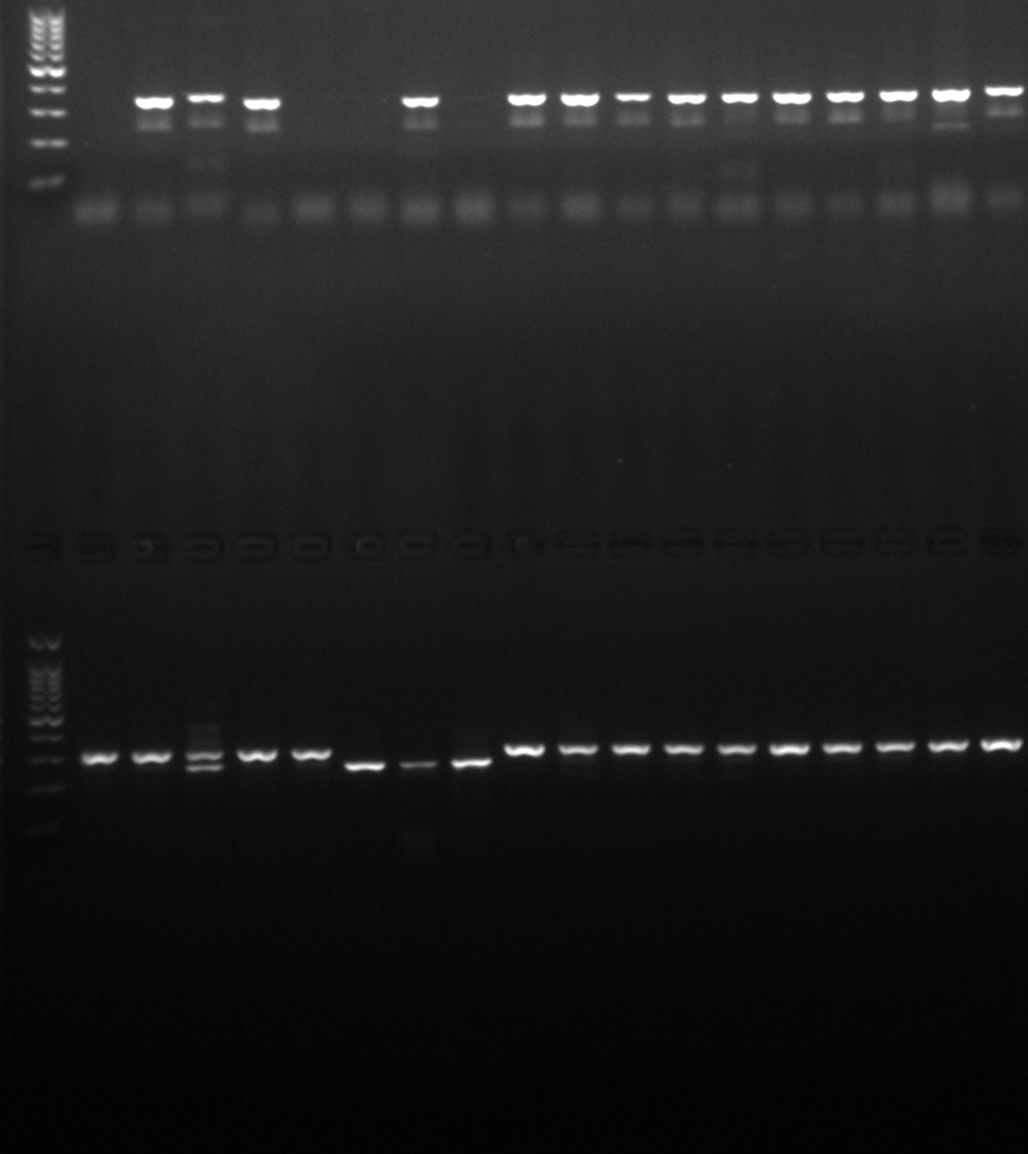
**

*FSF-R26^CAG−CreERT2.^* 350bp

*flx/flx flx/flx flx/+ flx/flx flx/flx +/+ +/+ +/+ flx/flx flx/flx*

**
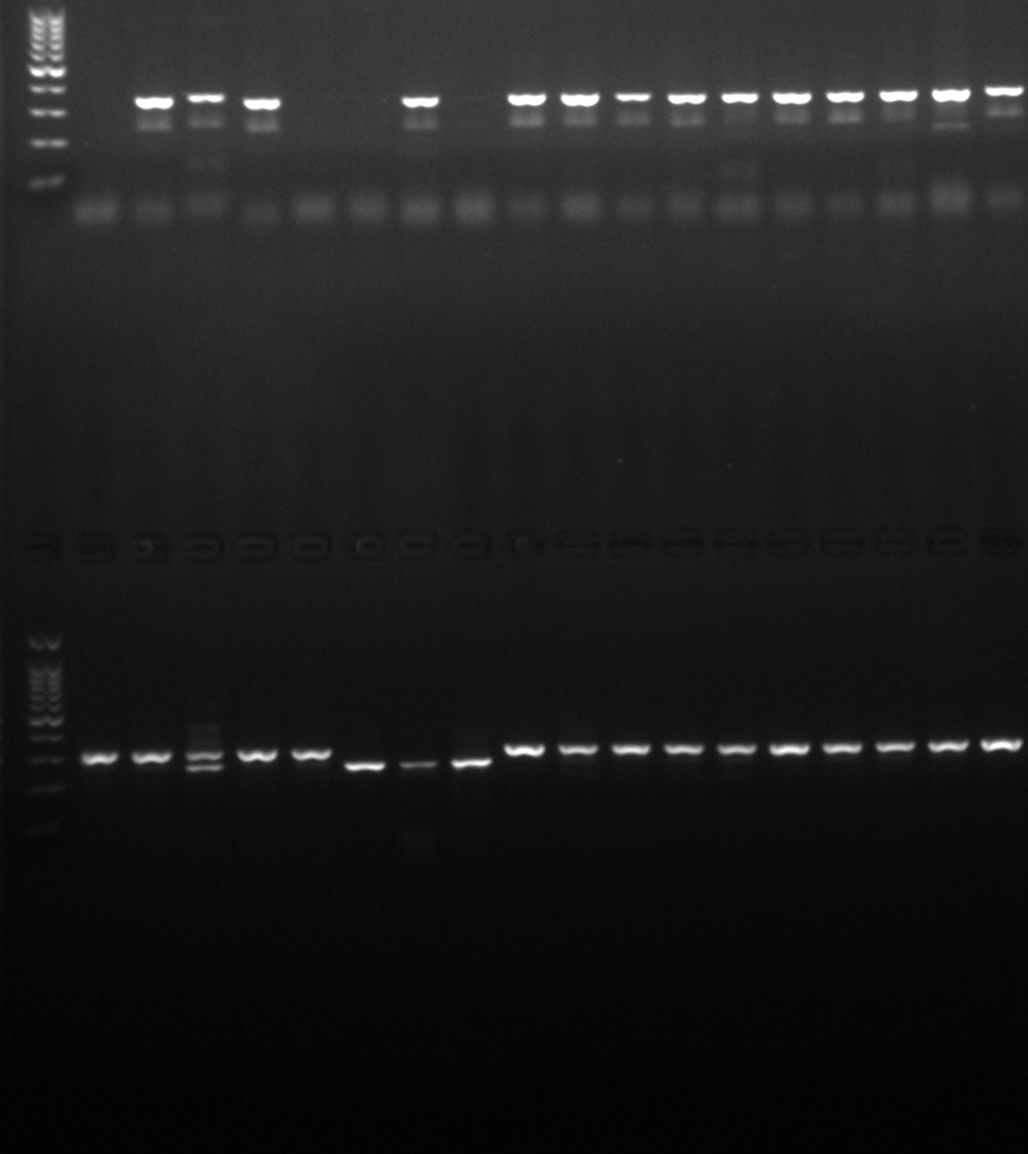
**

**
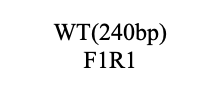
**

PSMD11^flx^(310bp)

M

Control

c

Vehicle

TAM

**
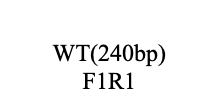

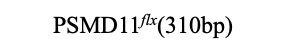
**
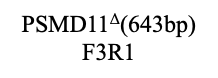
**
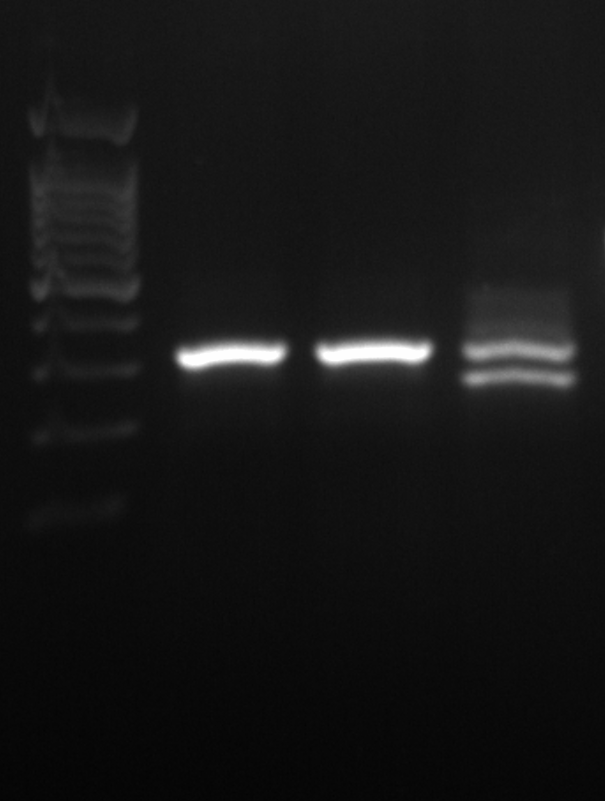

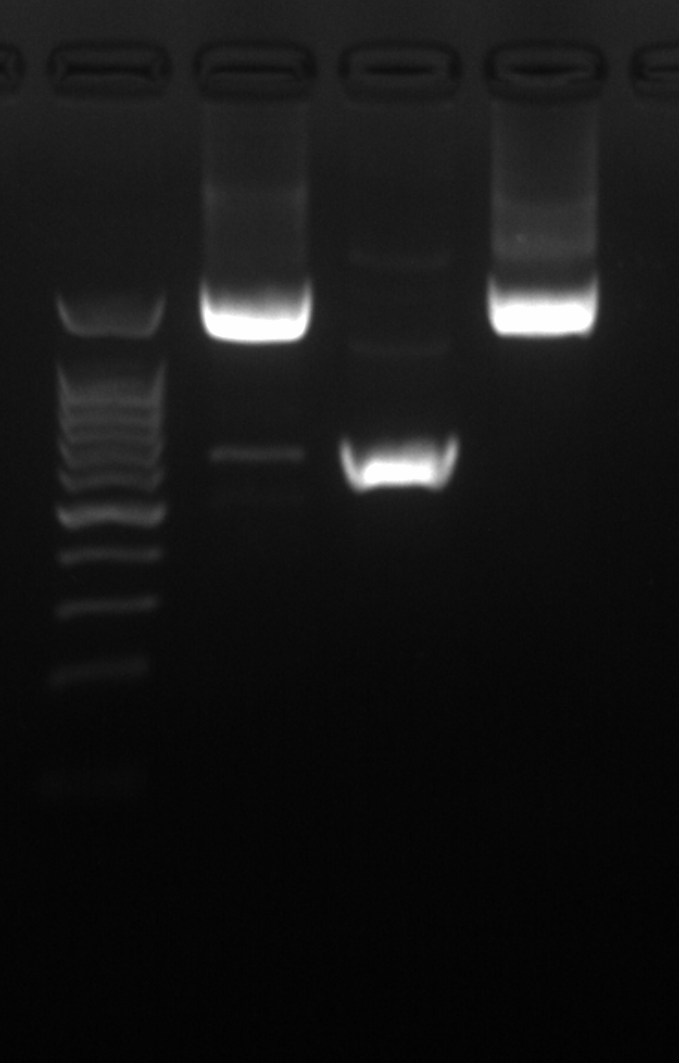
**

PSMD11*^flx/flx^* (1722bp)

**f**

TAM

Vehicle 2 3 4 5 days

TAM

Vehicle


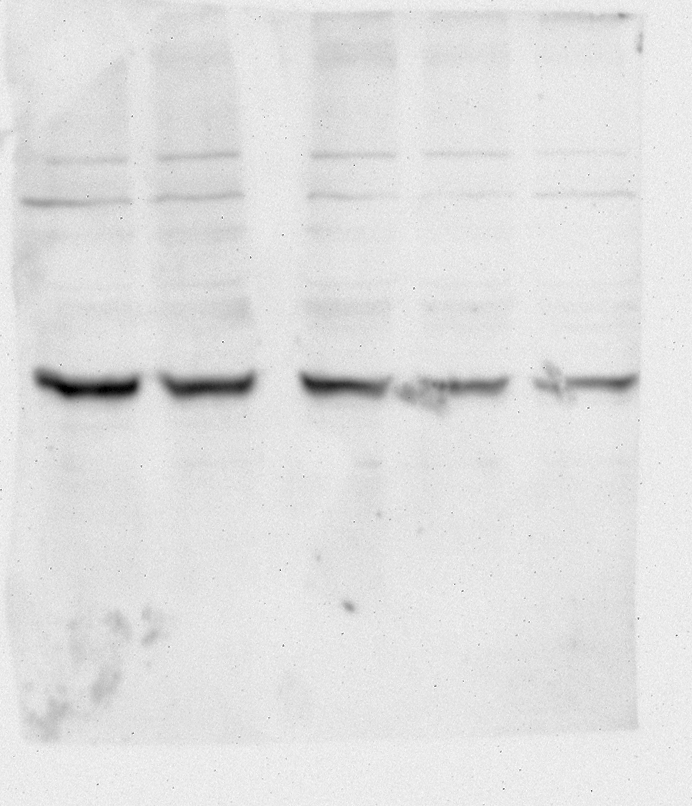


2 5 2 3 4 5 days


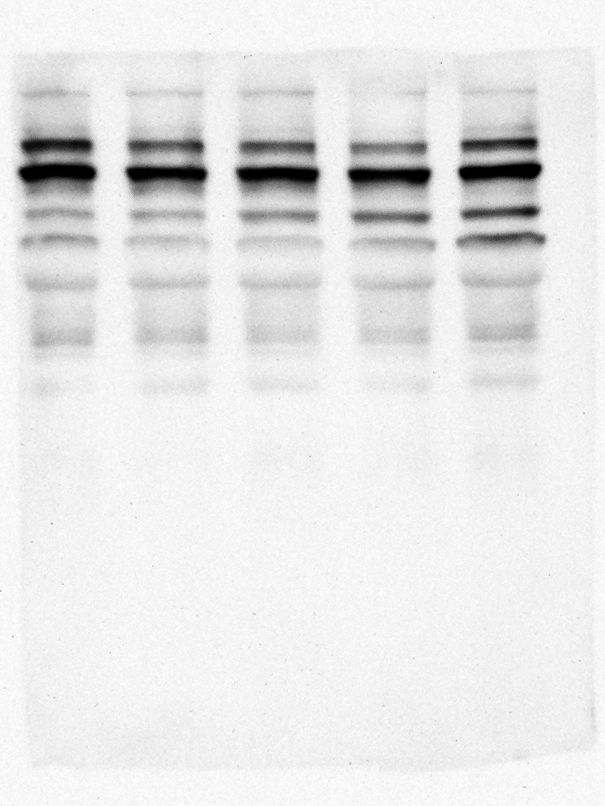
**
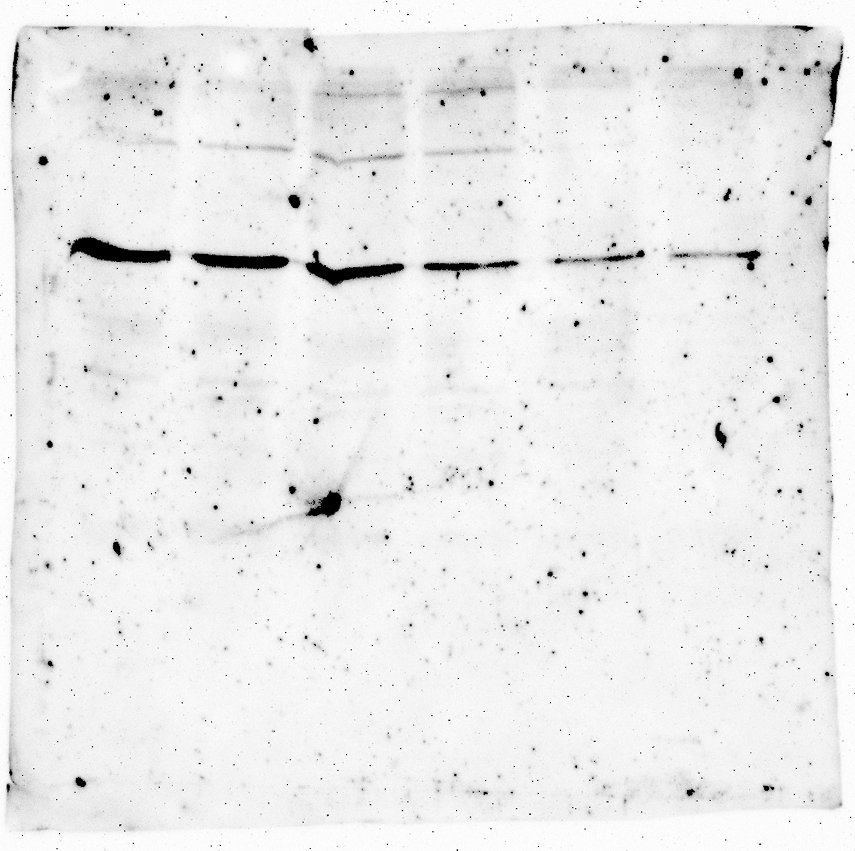
**

PSMD11

PSMD11


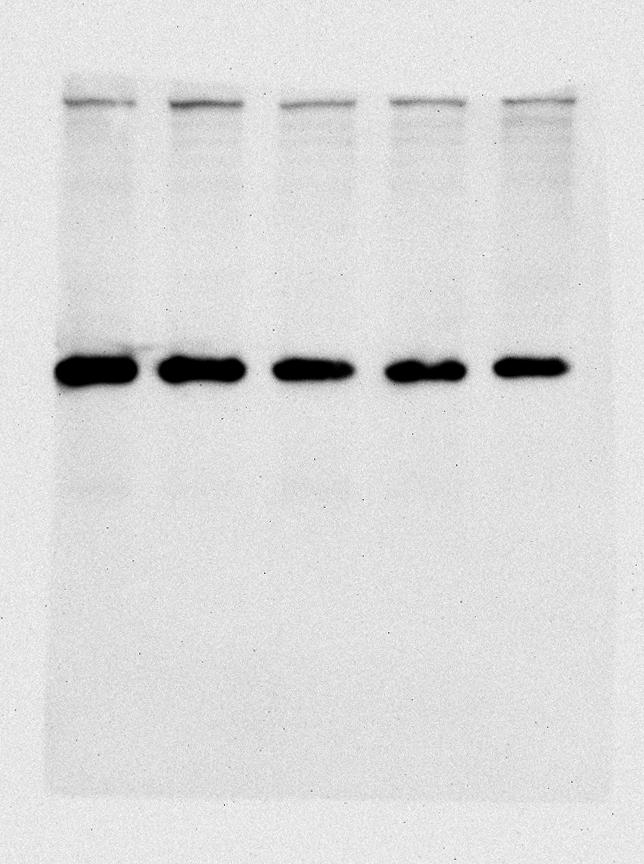
**
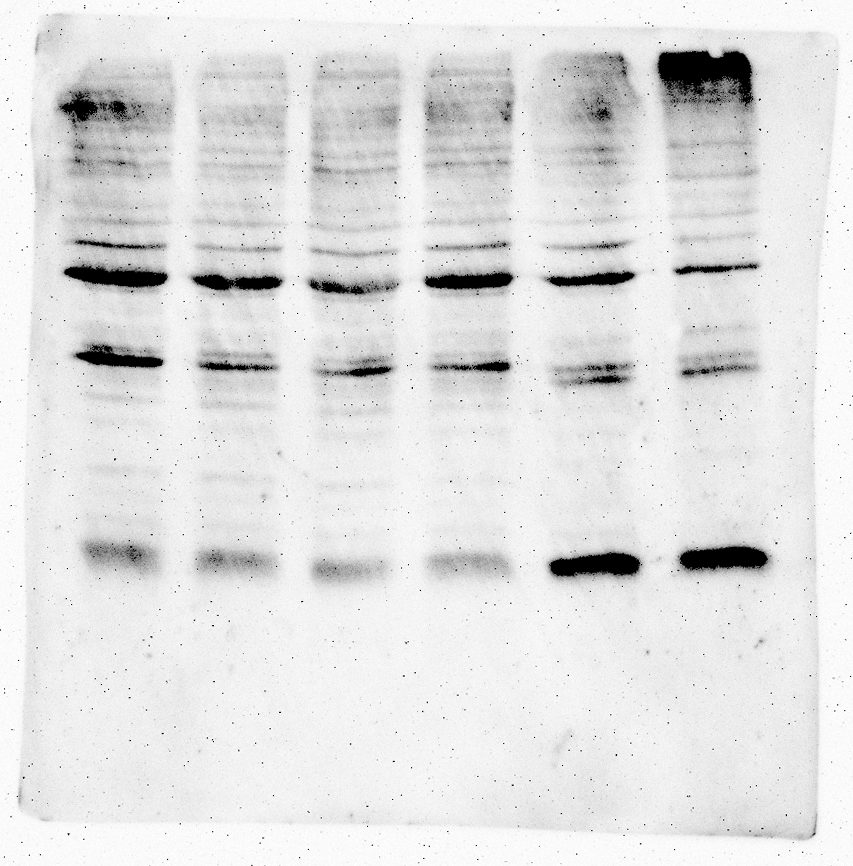
**

cleaved-Caspase 3

β-Actin

cleaved-PARP

**
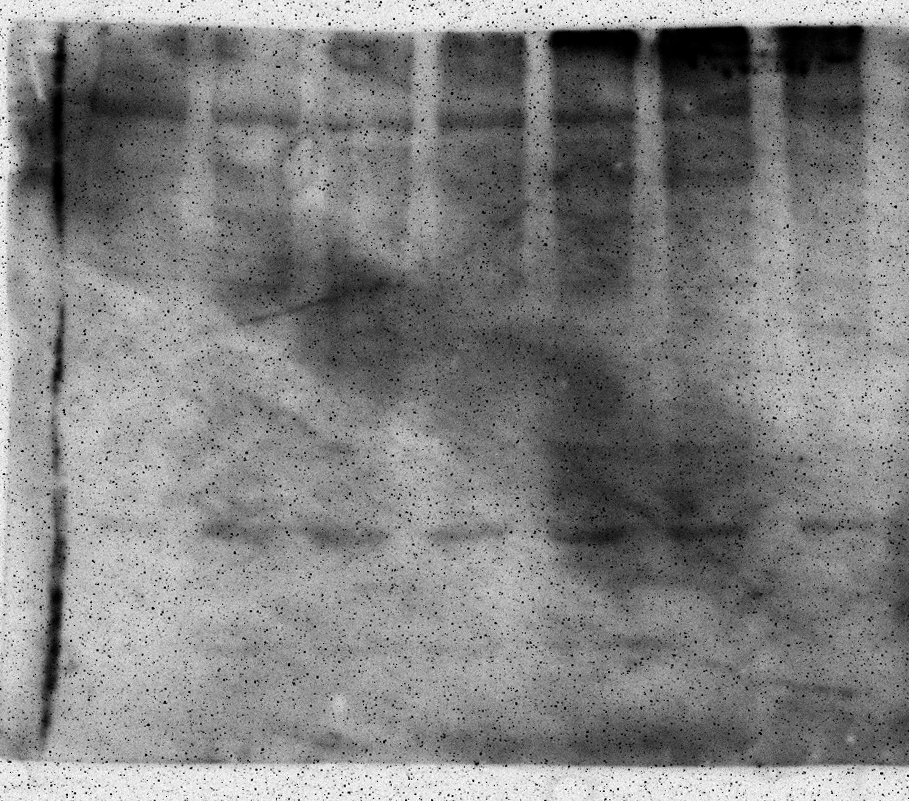
**

**
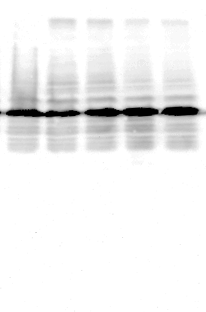
**

PSMD4

i

Pancreas Liver Kidney

WT *flx/flx* WT*flx/flx* WT *flx/flx*

**
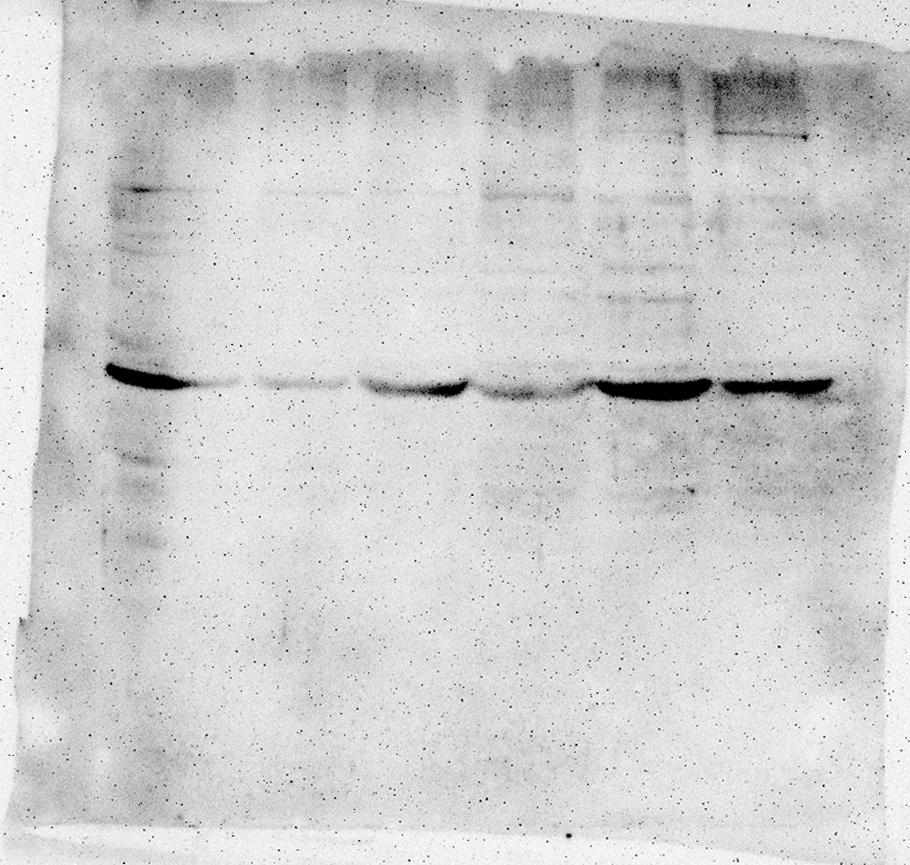
**

PSMD11

**
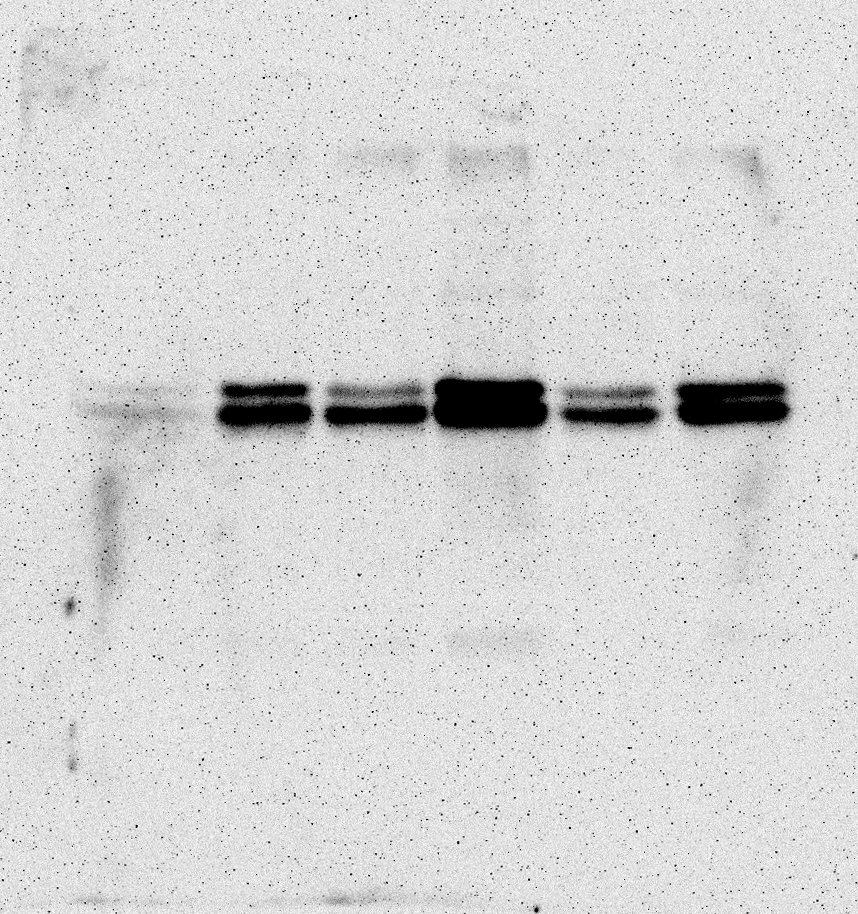
**

p53

**
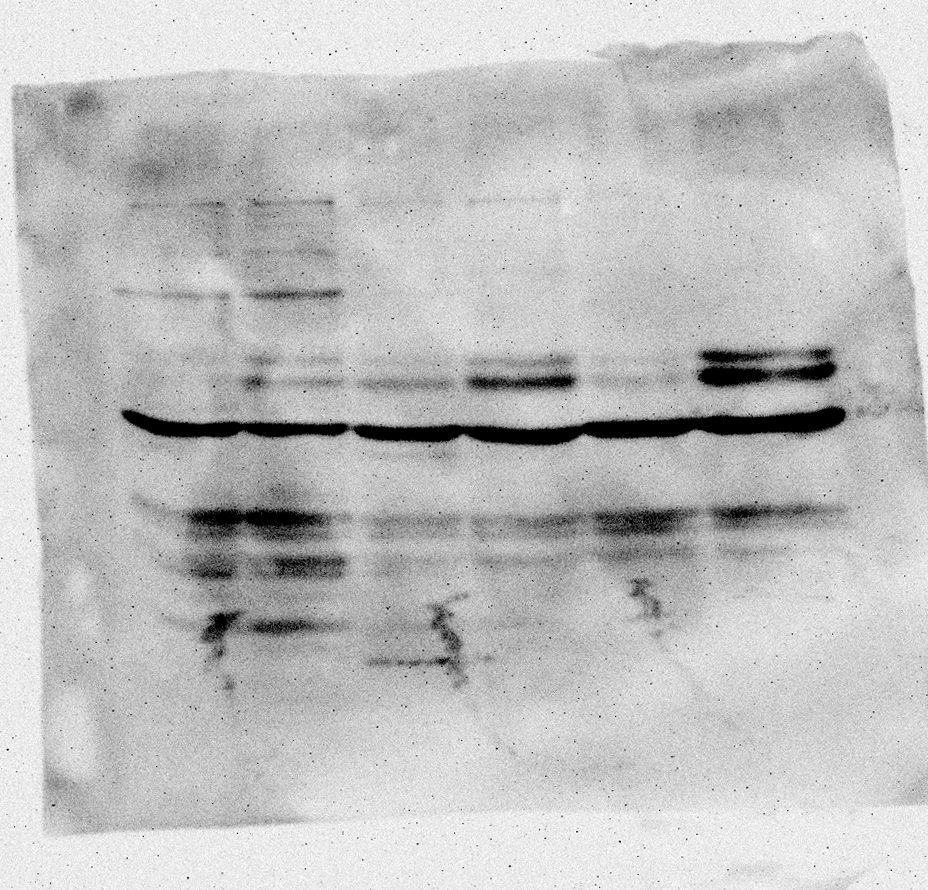
**

β-Actin

**Figure 5.** PSMD11 could be conditionally depleted in vitro and in vivo and could induce massive apoptosis in MEFs. **a** Genetic strategy to knockout PSMD11 by tamoxifen-mediated CreER^T2^ activation. **b** Genotyping of DNA from E14.5 embryos from intercrossing between *Flp;FSF-R26^CAG−CreERT2/+^*;*PSMD11 ^flx^*^/^*^+^* mice. Sizes of WT and mutant PCR bands are indicated. **c-e** MEFs were isolated by a standard protocol from E14.5 embryos with the genotype of *Flp;FSF-R26^CAG−CreERT2/+^*;PSMD11*^flx/flx^*, then the cells were treated for 5 days with 0.5uM 4-hydroxytamoxifen or vehicle (ethanol) to induce knockout of PSMD11, then Genotyping (**c**), immunofluorescence (**d**) and qRT-PCR (**e**) were performed to detect the change of expression of PSMD11. Control DNA was from a nonrecombined PSMD11 *^flx/+^* mouse without Cre expression, nuclei are counterstained with DAPI (blue) in immunofluorescence staining. Scale bars, 50 μm. **f** MEFs were treated with 0.5uM 4-hydroxytamoxifen or vehicle (ethanol) for the indicated times, then Western blotting was performed with antibodies directed against PSMD11, PSMD4, PARP, cleaved-Caspase 3, Ubiquitin, β-actin was used as loading control. **g** After treated with 0.5uM 4-hydroxytamoxifen or vehicle (ethanol) for 5 days, MEFs were stained with 1 μg/mL Hoechst 33342 for 20 min and imaged by confocal laser-scanning microscopy. Arrows denote apoptotic cells. **h** The chymotrypsin-like activity of proteasome was tested in 30 µg whole-cell extracts from vehicle or tamoxifen treated fibroblasts for 4 days with 0.1 mM Suc-LLVY-AMC peptide at 37°C for 180min in quadruplicates on a 96-well plate, Y-axis indicated the relative fluorescence units (RFU) reflecting the AMC cleavage from the peptide. Bars showed the mean±SD. **i** The FCP *^flx^*^/^*^flx^* and WT mice were fed with tamoxifen-containing food (400 mg/kg) or vehicle for 3 days, then the pancreas, liver and kidney were collected to detect the expression of PSMD11 and P53 with β-actin as loading control with Western blotting.

TAM

M Vehicle 2 3 4 5 days

**
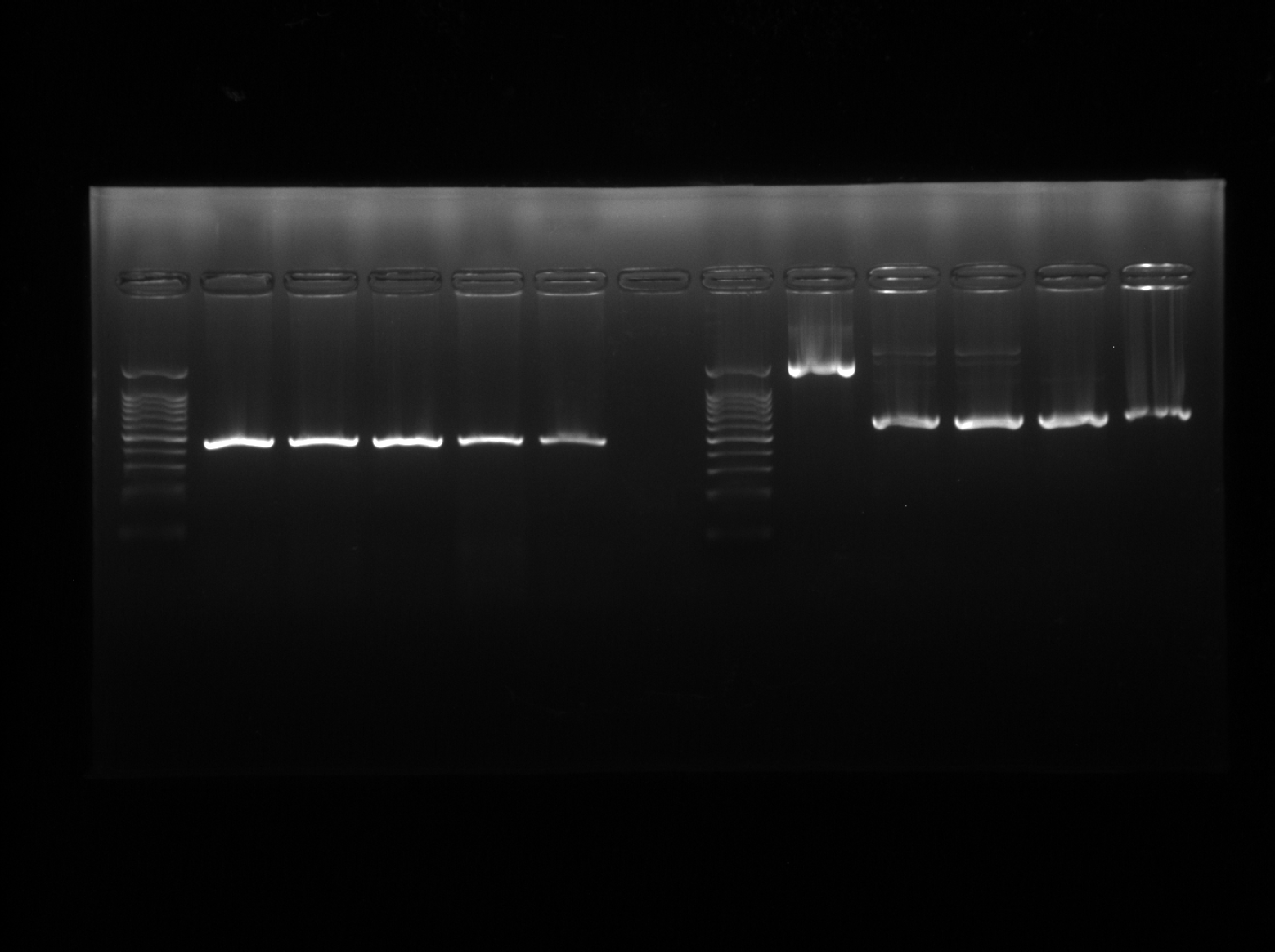
**

**
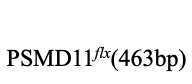
**

500

400

**
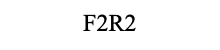
**

**
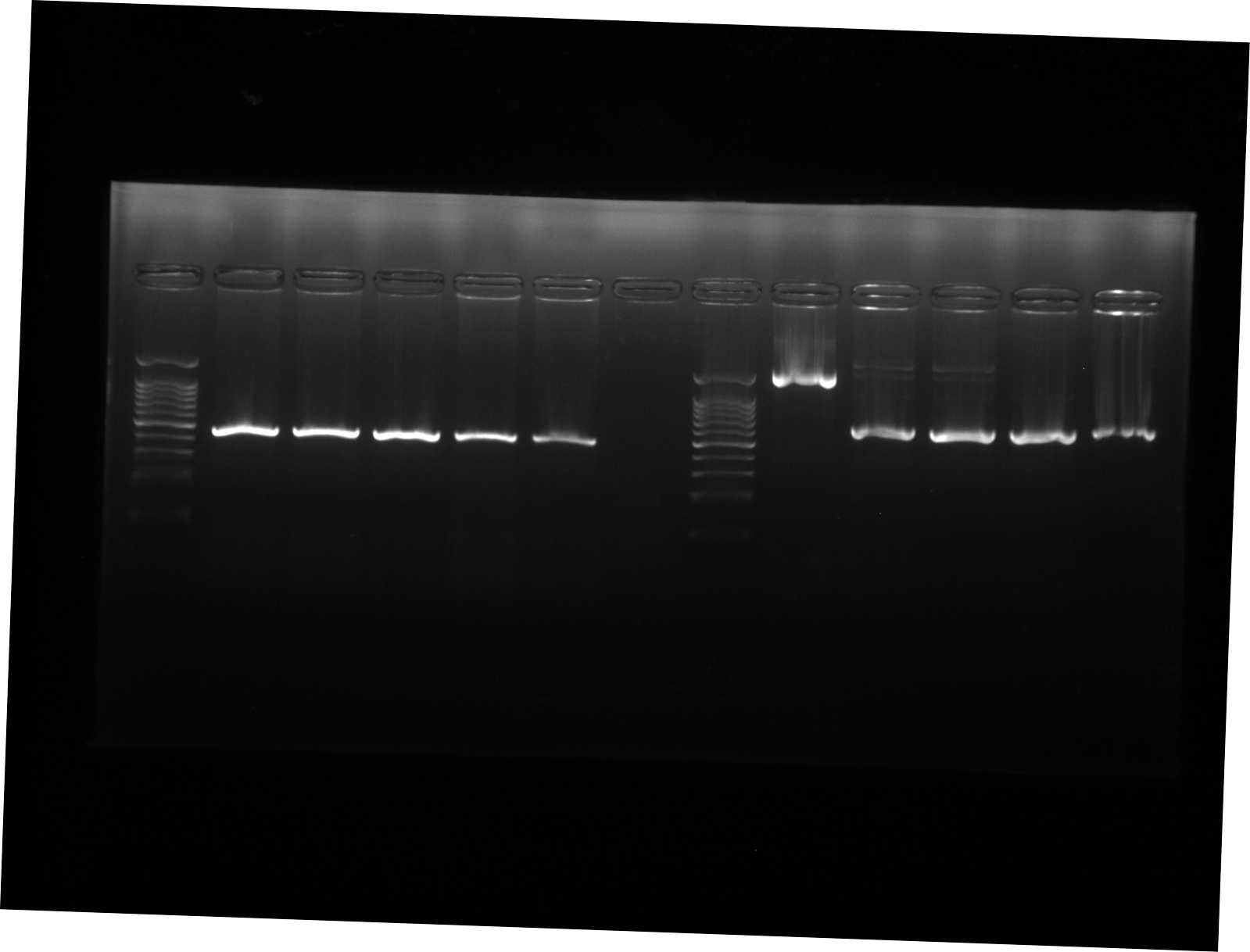
**

PSMD11*^flx/flx^* (1722bp)

700

**
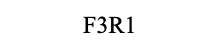

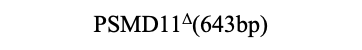
**

**Figure 6.** Tamoxifen could induce efficient deletion of the floxed allele of PSMD11 in MEFs with the genotype of *Flp;FSF-R26^CAG−CreERT2/+^*;PSMD11*^flx/flx^*. DNA was extracted from MEFs with the genotype of *Flp;FSF-R26^CAG−CreERT2/+^*;PSMD11*^flx/flx^*. treated for 5 days with 0.5uM 4-hydroxytamoxifen or vehicle (ethanol), PCR genotyping was performed using the primers as indicated, PCR bands were separated by 1% agarose gel electrophoresis. primer F3 and R1 will produce 643bp bands in PSMD11 exon 5 deleted alleles, primers F2 and R2 will produce 463bp and 335bp bands in PSMD11 exon 5 floxed or WT alleles respectively.
